# Activating GPR55 protects cochlear hair cells against cisplatin-induced ototoxicity via inhibiting MAPK pathway

**DOI:** 10.1038/s41598-026-48548-6

**Published:** 2026-04-14

**Authors:** Lingna Guo, Ruitang Wang, Yatang Wang, Houqiang Ruan, Yuhua Zhang, Yongjie Wei, Jiawei Du, Qiaojun Fang, Jianming Yang, Wei Cao

**Affiliations:** Department of Otolaryngology-Head and Neck Surgery, The Second Affiliated Hospital of Anhui Medical University, Hefei, 230601 China

**Keywords:** GPR55, O-1602, Ototoxicity, Hearing loss, Cisplatin, Cell biology, Drug discovery

## Abstract

Cisplatin (CDDP) is an effective chemotherapeutic agent used to treat solid tumors, but it can cause irreversible hearing loss. Currently, there are no specific preventive measures available for this side effect. G protein-coupled receptor 55 (GPR55) exhibits antioxidant, anti-inflammatory, and anti-apoptotic properties and is implicated in various disease processes. Nevertheless, whether GPR55 plays a role in CDDP-induced hearing loss remains unclear. We explored the effects and mechanisms of O-1602, a GPR55 agonist, on CDDP-induced ototoxicity. Our results showed that GPR55 is present in cochlear hair cells and HEI-OC1 cells, with increased expression following CDDP exposure. Moreover, O-1602-induced activation of GPR55 markedly mitigated the ototoxic effects of CDDP in HEI-OC1 cells, cochlear explants, and mouse models by preventing oxidative stress and apoptosis. In addition, GPR55 protected against CDDP-induced damage via inhibiting the MAPK pathway. Therefore, GPR55 is a potential therapeutic target for preventing CDDP-induced ototoxicity.

**Supplementary Information:**

The online version contains supplementary material available at 10.1038/s41598-026-48548-6.

## Introduction

Hearing impairment affects approximately 5% of the global population and represents a serious global health concern^1^. Many factors contribute to auditory impairment, such as aging, genetic predisposition, excessive noise exposure, and exposure to ototoxic chemicals^2–4^. Cisplatin (CDDP) is a chemotherapeutic drug commonly used for the treatment of solid tumors and is known to be ototoxic^5,6^. CDDP-induced ototoxicity affects various structures and cell types within the cochlea, with hair cells (HCs) being particularly vulnerable^7^. A key mechanism underlying CDDP-related ototoxicity is the overaccumulation of reactive oxygen species (ROS) in HCs, which activates apoptotic cascades that cause cell death^8–10^. Sodium thiosulfate has recently received FDA approval specifically for preventing hearing loss in pediatric patients treated with CDDP. However, significant clinical needs remain unmet concerning its use in adult patients and other specific populations or clinical scenarios. Therefore, novel therapeutic targets must be identified, and innovative pharmacological interventions should be developed to mitigate CDDP-related hearing loss.

G protein-coupled receptors (GPCRs) are widely distributed and play crucial roles in nearly all biological functions. They represent one of the largest classes of drug targets, accounting for 36% of all authorized medications that target these receptors^11^. Over 50 GPCRs have been identified within the inner ear, where they play roles in determining cell fate, promoting cell survival, and protecting HCs from ototoxic stress-induced damage^12,13^. For example, Adenosine A1 receptor (A1AR) activation protects the cochlea from CDDP-induced hearing loss via inhibiting oxidative stress and inflammatory pathways^14^. Additionally, A1AR protects against age-related and noise-induced hearing loss^15,16^. Cannabinoid receptor 2 (CB2R) is present in spiral vessels, spiral ganglion neurons, and HCs in the inner ear, and its expression increases following CDDP administration^17,18^. Moreover, CB2R activation can alleviate HC injury, prevent ribbon synapse degeneration, and mitigate hearing loss caused by CDDP^17,19^. Consequently, GPCRs are candidates for mitigating the ototoxic effects associated with CDDP exposure.

G protein-coupled receptor 55 (GPR55) is a nonclassical cannabinoid receptor with a strong binding affinity for various endogenous and synthetic cannabinoids, such as cannabidiol, anandamide, and O-1602^20–22^. L-α-lysophosphatidylinositol is a bioactive lipid that also activates GPR55^23,24^. Additionally, GPR55 is a key target of curcumin and contributes to its physiological effects^25^.

GPR55 is widely distributed across various organs and tissues, playing a vital role in numerous physiological processes^26^. It is also involved in several pathological conditions. Notably, GPR55 is a receptor located in brain regions associated with depression, with its levels significantly reduced in the medial orbitofrontal cortex of individuals exhibiting anxiety-like behaviors compared to healthy controls. Furthermore, activation of GPR55 with O-1602 reduces depression-associated symptoms^27^. L-α-lysophosphatidylinositol demonstrates neuroprotective effects following injury by activating the GPR55 pathway to mitigate cellular inflammation and oxidative stress^28,29^. Moreover, O-1602 mitigates cognitive impairments and neurotoxicity in Alzheimer’s disease models, indicating that GPR55 is a promising therapeutic target for this condition^30^. These results highlight the importance of GPR55 in preserving physiological functions and its potential as a therapeutic target for various disorders.

The function of GPR55 in sensorineural hearing loss remains unclear despite extensive research. This study revealed that GPR55 is localized in the HCs of the auditory system, with its expression levels increasing in response to CDDP-induced ototoxicity. Moreover, the functionality of HEI-OC1 cells and HCs, which was impaired by CDDP treatment, improved following pretreatment with O-1602, indicating a protective role for GPR55. Thus, GPR55 represents a potential therapeutic target for combating the ototoxic effects of CDDP exposure.

## Materials and methods

### Compounds

CDDP (MCE, HY-17394) was dissolved in N, N-Dimethylformamide (DMF). O-1602 and ML-193, purchased from MCE (HY-107541 and HY-110125, respectively), were dissolved in dimethyl sulfoxide (DMSO) at their respective storage concentrations. These compounds were diluted to the appropriate concentrations in tissue culture medium or high-glucose Dulbecco’s Modified Eagle Medium (DMEM) for treating tissues or cells.

### Cell culture

HEI-OC1 cells, purchased from Cyagen (Guangzhou, China), were grown in DMEM supplemented with 10% fetal bovine serum to provide suitable growth conditions. The cells were maintained at 33 °C in a humidified environment containing 5% CO_2_. A solution of 0.25% trypsin with EDTA was used to detach the cells from the surface.

### Mouse model

We used six-week-old male C57BL/6 mice, which were obtained from GemPharmatech (Nanjing, China), to develop an in vivo model of CDDP-induced sensorineural hearing loss. All experiments involving animals were performed in accordance with the directives established by the Hefei Comprehensive National Science Center’s Health Experimental Animal Welfare and Ethics Committee with the approval number IHM-AP-2024-032 and were conducted in strict adherence to the ARRIVE guidelines. All mice were housed under controlled environmental conditions, maintaining optimal humidity and temperature, with a 12-hour (h) light/dark cycle and access to ample water and food. After confirming normal hearing function through auditory brainstem response (ABR) testing, the mice were randomly grouped into several groups: a control group, a furosemide (FO) treatment group, an O-1602 treatment group, a CDDP treatment group, and a combined treatment group receiving both O-1602 and CDDP. The O-1602 group was given an injection of 5 mg/kg two hours beforehand, while the other groups were administered equivalent doses of DMSO. Two hours later, FO (200 mg/kg) was administered to promote CDDP-induced ototoxicity, followed by CDDP at 0.6 mg/kg after a 30-minute delay. Following drug administration, each mouse received a 500 µL injection of 0.9% saline to minimize discomfort and mortality throughout the procedure. All treatments were delivered via intraperitoneal injection to ensure the effective systemic absorption of the treatments. Hearing condition was assessed 7 days after administration. After the ABR test, the mice were humanely euthanized using CO_2_, and their cochleae were dissected for analysis of HCs and synapses.

### Cochlear explant culture

The cochleae were carefully collected from mice on postnatal day 3 (P3). Surrounding tissue was removed in cold HBSS to ensure that the cochleae were clean and ready for further experimentation. The isolated cochlear explants were placed on sterilized glass coverslips precoated with Cell-Tak. These coverslips were placed in four-well plates to create the culture environment. The explants were then cultured in DMEM enriched with N2 and B27 (Gibco, 17502048 and 17504044, respectively). The entire culture setup was maintained at 37 °C in a 5% CO_2_ environment within a cell incubator for overnight incubation.

### Cell viability

Cell viability was assessed using a Cell Counting Kit-8 (CCK-8; Beyotime, C0038). HEI-OC1 cells were plated in 96-well plates and allowed to incubate overnight before treatment. The original medium was removed from each well after treatment and substituted with 100 µL of DMEM containing 10 µL of CCK-8 reagent. The cells were then incubated for 2 h before being examined.

### Protein extraction and Western blotting

Samples of cells or tissues were gathered and lysed using RIPA buffer containing 1% protease inhibitor cocktail. The extracted proteins were analyzed using polyacrylamide gel electrophoresis, for which equal amounts of protein were separated. The proteins were transferred onto polyvinylidene fluoride membranes after electrophoresis. The membranes were blocked at room temperature (RT) using 5% skim milk. We incubated the polyvinylidene fluoride membranes at 4 °C overnight. The primary antibodies utilized in this procedure are listed in Supplementary Table 1. The secondary antibodies were subsequently incubated with the membranes for 2 h at RT, and an ECL detection kit was used to detect the protein bands.

### RNA extraction and qPCR

The cells were harvested after treatment, and the total RNA extraction was carried out using TRIzol reagent in accordance with the provided instructions. Complementary DNA (cDNA) was synthesized, and qPCR was conducted on a Bio-Rad CFX96 real-time PCR system with the primers listed as follows.

**Table:** 

Primer name	Primer sequences (Forward)	Primer sequences (Reverse)
Caspase-3	ATGGAGAACAACAAAACCTCAGT	TTGCTCCCATGTATGGTCTTTAC
Caspase-8	TGCTTGGACTACATCCCACAC	TGCAGTCTAGGAAGTTGACCA
Apaf-1	AGTAATGGGTCCTAAGCATGTTG	GCGATTGGGAAAATCACGTAAAA
FADD	GCGCCGACACGATCTACTG	TTACCCGCTCACTCAGACTTC
Bcl-2	ATGCCTTTGTGGAACTATATGGC	GGTATGCACCCAGAGTGATGC
GAPDH	AGGTCGGTGTGAACGGATTTG	TGTAGACCATGTAGTTGAGGTCA

### Immunofluorescence staining

We used 4% paraformaldehyde to fix the cells and tissues at RT for 1 h. Permeabilization was performed by incubating the samples in 0.5% PBST for 30 min, followed by a blocking step using 10% donkey serum for one hour. The samples were then incubated overnight at 4 °C in the presence of primary antibodies (see Supplementary Table 1). Afterward, the cells or tissues were rinsed three times with PBS, each wash lasting for 5 min, and incubated with secondary antibodies at RT for 2 h. Finally, the samples were treated with DAKO and mounted on glass coverslips. Images were acquired using a fluorescence microscope (Carl Zeiss).

### ROS detection

The levels of ROS in both HCs and HEI-OC1 cells were assessed using mtSOX Deep Red (Dojindo Laboratories, MT14). The samples were washed with PBS and subsequently incubated at 37 °C in prewarmed DMEM containing 10 µM mtSOX Deep Red, kept out of light. Afterward, the samples were washed three times with PBS for 5 min each. Fluorescent images were captured using a Carl Zeiss confocal fluorescence microscope.

### ABR test

The mice were sedated with an intraperitoneal injection of pentobarbital sodium at a dose of 100 mg/kg and gently placed into a sound-insulated chamber. During anesthesia, the mice were positioned on a heating pad set to 37 °C to maintain their body temperature and minimize potential discomfort or mortality. A recording electrode was subcutaneously inserted at the midline of the cranial roof, while the reference electrode was positioned behind the ear. We used a TDT System III (Tucker-Davis Technologies, USA) to record all responses, using brief pure tones as stimuli. The ABR thresholds and wave I amplitudes of the mice were measured at 4, 8, 12, 16, 24, and 32 kHz.

### RNA sequencing (RNA-seq)

The total RNA was extracted using TRIzol Reagent. The purity, concentration, and RNA integrity number of the isolated RNA samples were assessed to ensure their quality and suitability for further analysis. The cDNA library was sequenced using the Illumina HiSeq platform. The cDNA expression levels of all samples were compiled into an expression matrix, and differential expression was analyzed at the gene or transcript level to identify functional differences related to sample grouping using DESeq2^6^ software. Statistical significance was set at a P-value < 0.05 and a fold change in expression of 2 or above (|log2FC| ≥ 1).

### Statistical analysis

Data analysis was conducted using GraphPad Prism, and the results were presented as the mean ± standard error of the mean (SEM). One-way ANOVA was employed to identify differences among groups, while a t-test was used to determine significant differences between groups. Statistical significance is indicated by asterisks as follows: ^*^P < 0.05, ^**^P < 0.01, ^***^P < 0.001, and ^****^P < 0.0001.

## Results

### CDDP increased GPR55 levels in HEI-OC1 cells and cochlear explants

We thoroughly explored the role of GPR55 in CDDP-induced ototoxicity. Initially, GPR55 expression was detected using Myosin7a as a marker for HCs. Our immunostaining results revealed that GPR55 was present in the HCs of the mouse cochlea (Fig. S1A). Additionally, we detected GPR55 protein expression in HEI-OC1 cells (Fig. S1B), further confirming the presence of GPR55 in auditory cells.

**Fig. 1:**
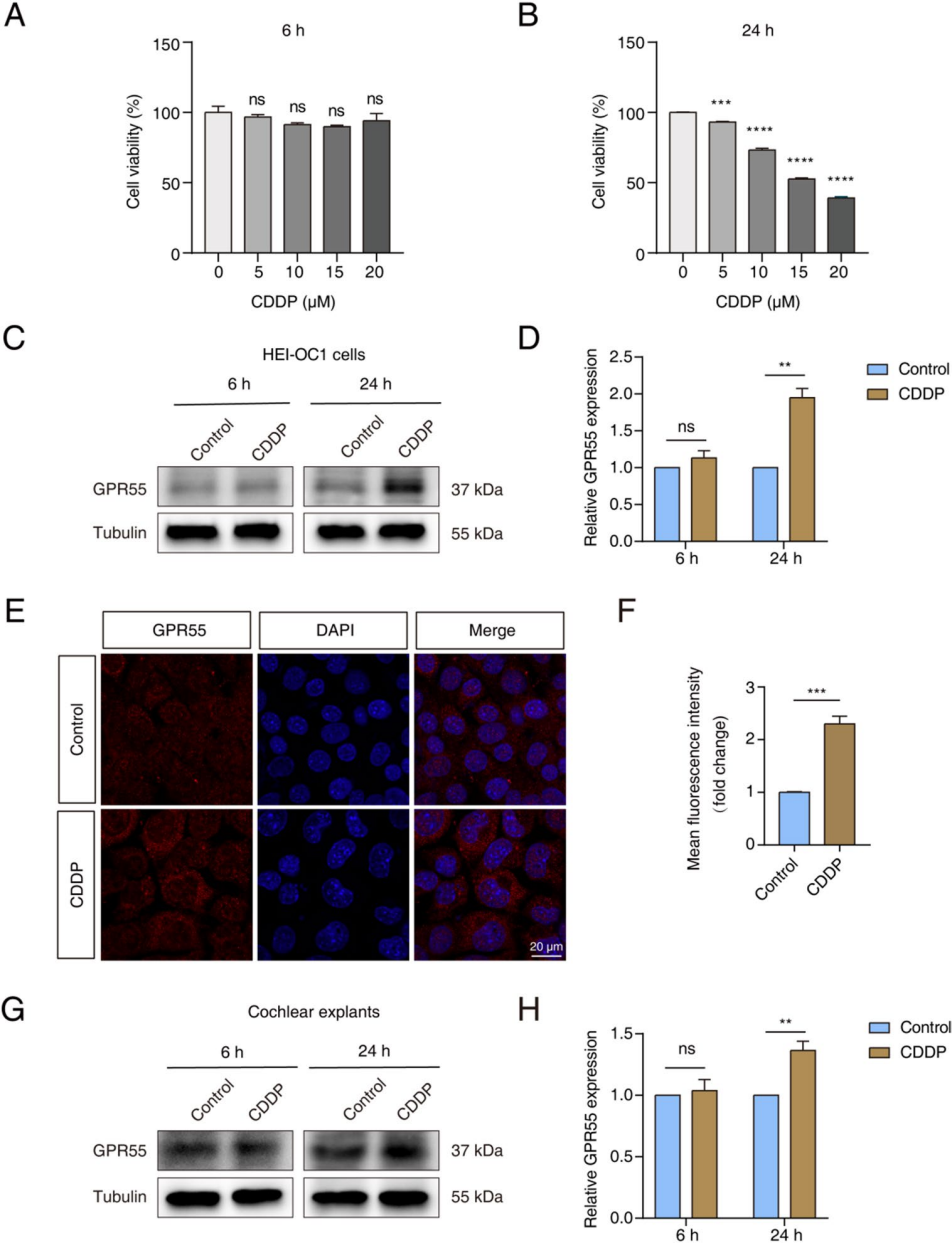
The levels of GPR55 were elevated in both HEI-OC1 cells and cochlear explants following CDDP exposure. (**A**,** B**) The CCK-8 assay assessed cell viability at various CDDP concentrations (0, 5, 10, 15, or 20 µM) after 6 or 24 h of exposure. (**C**,** D**) Representative western blots demonstrated the expression levels of GPR55 in HEI-OC1 cells that were given 15 µM CDDP for 6 or 24 h. (**E**,** F**) Immunofluorescence staining showed the levels of GPR55 expression in HEI-OC1 cells from both the control group and the group treated with 15 µM CDDP. (**G**,** H**) Representative western blots depicted the expression levels of GPR55 in cochlear explants subjected to 150 µM CDDP for 6 or 24 h. h: hour; CDDP: cisplatin.

We then verified the protein levels of GPR55 following treatment of HEI-OC1 cells and cochlear explants with CDDP. We developed a CDDP-induced injury model in HEI-OC1 cells by exposing the cells to various concentrations of CDDP (0, 5, 10, 15, or 20 µM) for either 6 or 24 h. The CCK-8 assay showed that cell survival decreased in a dose-dependent manner after 24 h, with cell viability dropping to about 50% at 15 µM CDDP. However, no cell damage was observed after 6 h of treatment (Fig. 1A and B). Next, we measured GPR55 expression levels using Western blotting and immunostaining. Western blot analysis depicted that GPR55 protein levels increased after 24 h of exposure to 15 µM CDDP compared to the control, with no significant difference observed after 6 h of exposure (Fig. 1C and D). Immunostaining results supported these findings, showing that GPR55 protein levels were markedly elevated after 24 h of treatment with 15 µM CDDP (Fig. 1E and F).

We also investigated GPR55 levels in cultured cochlear explants to better understand its role in the cellular response to damage. The optimal concentration of CDDP for the injury model was determined by treating cochlear tissues harvested from P3 mice with 0, 50, 100, or 150 µM CDDP for 24 h. Treatment with 150 µM CDDP caused severe damage to HCs, as evidenced by a marked decrease in Myosin7a-positive cells across the basal, middle, and apical turns of the cochlea compared to the control group. In contrast, no HC injury was observed at 50 or 100 µM (Fig. S2A-D). Therefore, we chose 150 µM CDDP for subsequent experiments. Western blot analysis revealed a marked increase in GPR55 protein levels after 24 h of CDDP treatment, while no notable change was observed at 6 h (Fig. 1G and H). Overall, these results indicate that GPR55 expression is substantially upregulated in HEI-OC1 cells and cochlear explants following CDDP exposure, suggesting that GPR55 is involved in CDDP-induced hearing impairment.

### GPR55 activation attenuated CDDP-induced apoptosis in HEI-OC1 cells

**Fig. 2:**
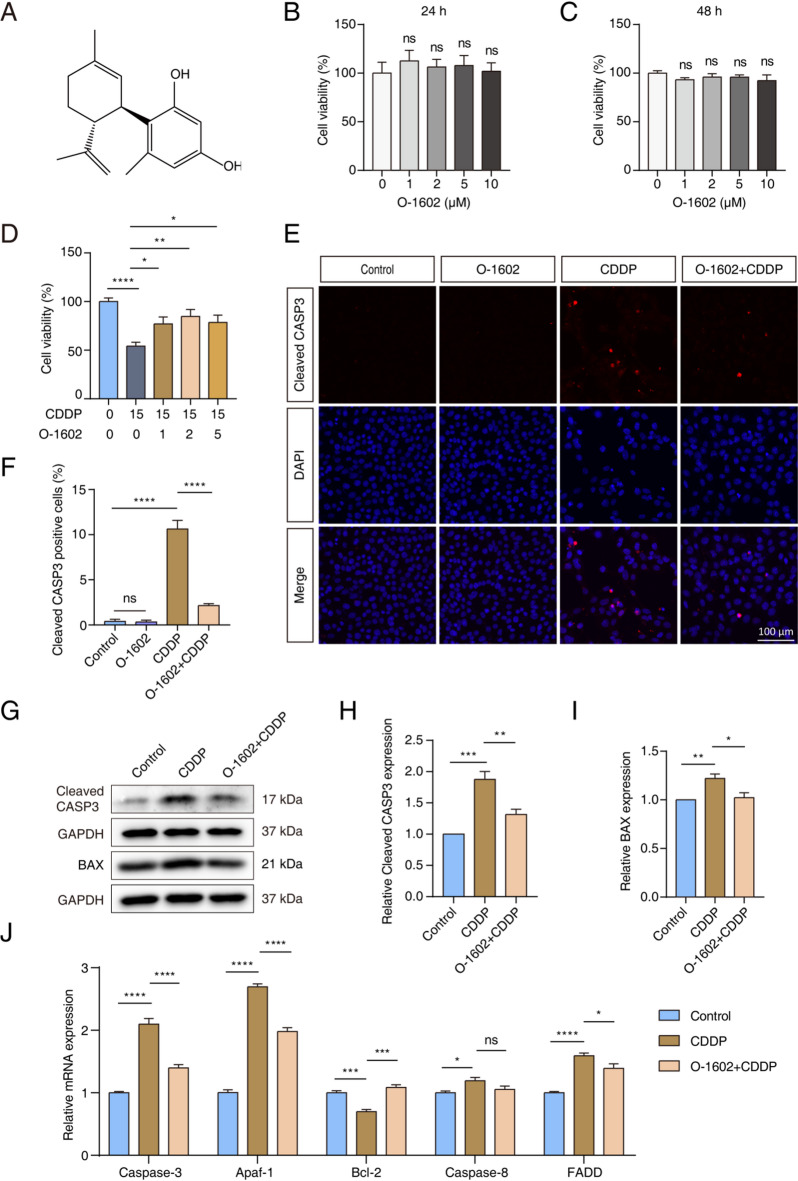
O-1602 reduced apoptosis induced by CDDP in HEI-OC1 cells. (**A**) Chemical structure of O-1602. (**B**,** C**) The CCK-8 assay indicated the cytotoxic effects of O-1602 at concentrations of 0, 1, 2, 5, or 10 µM on HEI-OC1 cells at 24 or 48 h. (**D**) The CCK-8 assay measured the viability of HEI-OC1 cells pretreated with O-1602 for 2 h, followed by exposure to 15 µM CDDP. (**E**,** F**) Representative images showed cleaved CASP3 staining in each group of HEI-OC1 cells. (**G-H**) Representative western blots illustrated the expression of cleaved CASP3 and BAX in HEI-OC1 cells among different groups. (**J**) qPCR results demonstrated the expression of genes associated with apoptosis. Cleaved CASP3: cleaved Caspase-3.

To investigate the specific function of GPR55, we utilized O-1602, a selective GPR55 activator^21^(Fig. 2A). We assessed the toxicity of O-1602 using a CCK-8 assay in HEI-OC1 cells. Cells were treated with O-1602 at concentrations of 0, 1, 2, 5, or 10 µM for 24 or 48 h. There was no significant change in cell viability across different concentrations or treatment durations, suggesting that O-1602 is not toxic to HEI-OC1 cells at concentrations up to 10 µM (Fig. 2B, C).

We then assessed the effect of O-1602 on ototoxicity induced by CDDP. HEI-OC1 cells underwent pretreatment with varying concentrations of O-1602 for 2 h, followed by co-incubation with 15 µM CDDP for 24 h. The CCK-8 assay revealed that cell viability was considerably lower in the group treated with 15 µM CDDP compared to the control group. However, cell viability was higher with O-1602 pretreatment than without, with 2 µM O-1602 providing the strongest protection against the toxic effects of CDDP (Fig. 2D). Therefore, we chose 2 µM O-1602 for use in subsequent experiments with HEI-OC1 cells.

We confirmed the protective function of GPR55 against CDDP-induced cellular damage by assessing apoptosis. Figure 2E and F demonstrate that the number of cleaved CASP3-positive cells was higher in the CDDP-treated group than in the control group, indicating that CDDP exposure triggered apoptosis in these cells. Pretreatment with O-1602 reduced the percentage of apoptotic cells compared to the group without pretreatment, highlighting the protective role of O-1602. Treatment with O-1602 alone did not adversely affect the HEI-OC1 cells. Western blot analysis further supported these findings, showing that O-1602 pretreatment diminished the levels of apoptosis-related proteins, cleaved CASP3 and BAX, compared to treatment with CDDP alone (Fig. 2G-I). Additionally, the qPCR results indicated that the expression levels of apoptosis-related and anti-apoptotic genes varied across different treatments. As shown in Fig. 2J, CDDP treatment increased the expression of apoptotic genes such as *Caspase-3*, *Caspase-8*, *Apaf-1*, and *FADD* compared to the control group. However, pretreatment with O-1602 considerably reduced the expression levels of *Caspase-3*, *Apaf-1*, and *FADD*. Although *Caspase-8* expression tended to decrease in the O-1602 pretreatment group compared to the group without pretreatment, this reduction was not statistically significant. In contrast, *Bcl-2* expression levels declined following CDDP treatment but increased after O-1602 pretreatment, further supporting the protective function of O-1602 against CDDP-induced apoptosis in HEI-OC1 cells.

### GPR55 activation alleviated CDDP-induced oxidative stress in HEI-OC1 cells

**Fig. 3:**
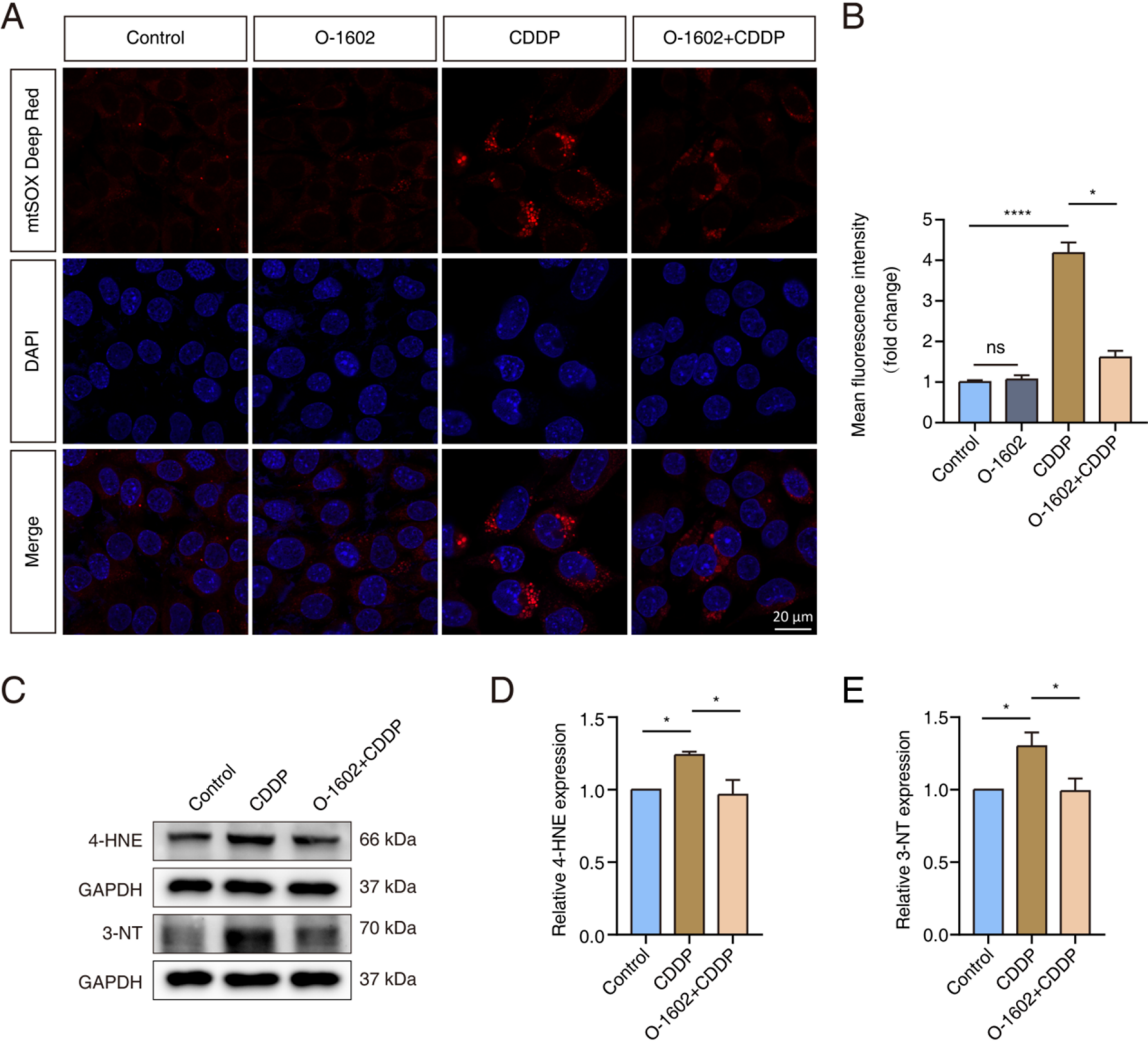
O-1602 alleviated oxidative stress caused by CDDP in HEI-OC1 cells. (**A**,** B**) Representative staining images showing mtSOX Deep Red (red) fluorescence in each experimental group of HEI-OC1 cells. (**C-E**) Western blot analysis of 4-HNE and 3-NT expression levels. 4-HNE: 4-Hydroxynonenal; 3-NT: 3-Nitrotyrosine.

The primary factor responsible for the ototoxic effects of CDDP is oxidative stress. Therefore, we assessed the effect of O-1602 on CDDP-induced oxidative stress in HEI-OC1 cells. We used the fluorescent probe mtSOX Deep Red to estimate mitochondrial superoxide levels. The fluorescence intensity of mtSOX Deep Red increased following exposure to CDDP. Importantly, this increase was markedly lower in cells pretreated with 2 µM O-1602 compared to cells treated with CDDP alone (Fig. 3A, B). Additionally, we measured the levels of 4-Hydroxynonenal (4-HNE) and 3-Nitrotyrosine (3-NT), which are indicators of oxidative stress, in HEI-OC1 cells to further validate our results^31^. Both 4-HNE and 3-NT expression levels increased following CDDP exposure but were reduced when the cells were pretreated with O-1602 (Fig. 3C-E). In general, activation of GPR55 with O-1602 diminishes CDDP-induced oxidative stress in HEI-OC1 cells.

We concurrently administered ML-193, a selective GPR55 antagonist^32^, along with O-1602 to validate the protective effects of O-1602 against CDDP-induced toxicity. We assessed the cytotoxicity of ML-193 on cells using a CCK-8 assay. ML-193 concentrations up to 20 µM were non-toxic to HEI-OC1 cells (Fig. S3A), confirming its safety for cellular applications. We then simultaneously treated HEI-OC1 cells with 10 µM ML-193 and 2 µM O-1602 following CDDP exposure. Subsequent Western blot analysis demonstrated that ML-193 treatment effectively abolished the protective effects of O-1602, as levels of cleaved CASP3 and 4-HNE did not reduce when both treatments were applied (Fig. S3B-D). Immunofluorescence analysis of cleaved CASP3 supported these findings (Fig. S3E, F), further validating our hypothesis that activation of GPR55 by O-1602 is essential for protecting HEI-OC1 cells from CDDP-induced ototoxicity.

### Activating GPR55 protected against CDDP-induced HC loss in cochlear explants

**Fig. 4:**
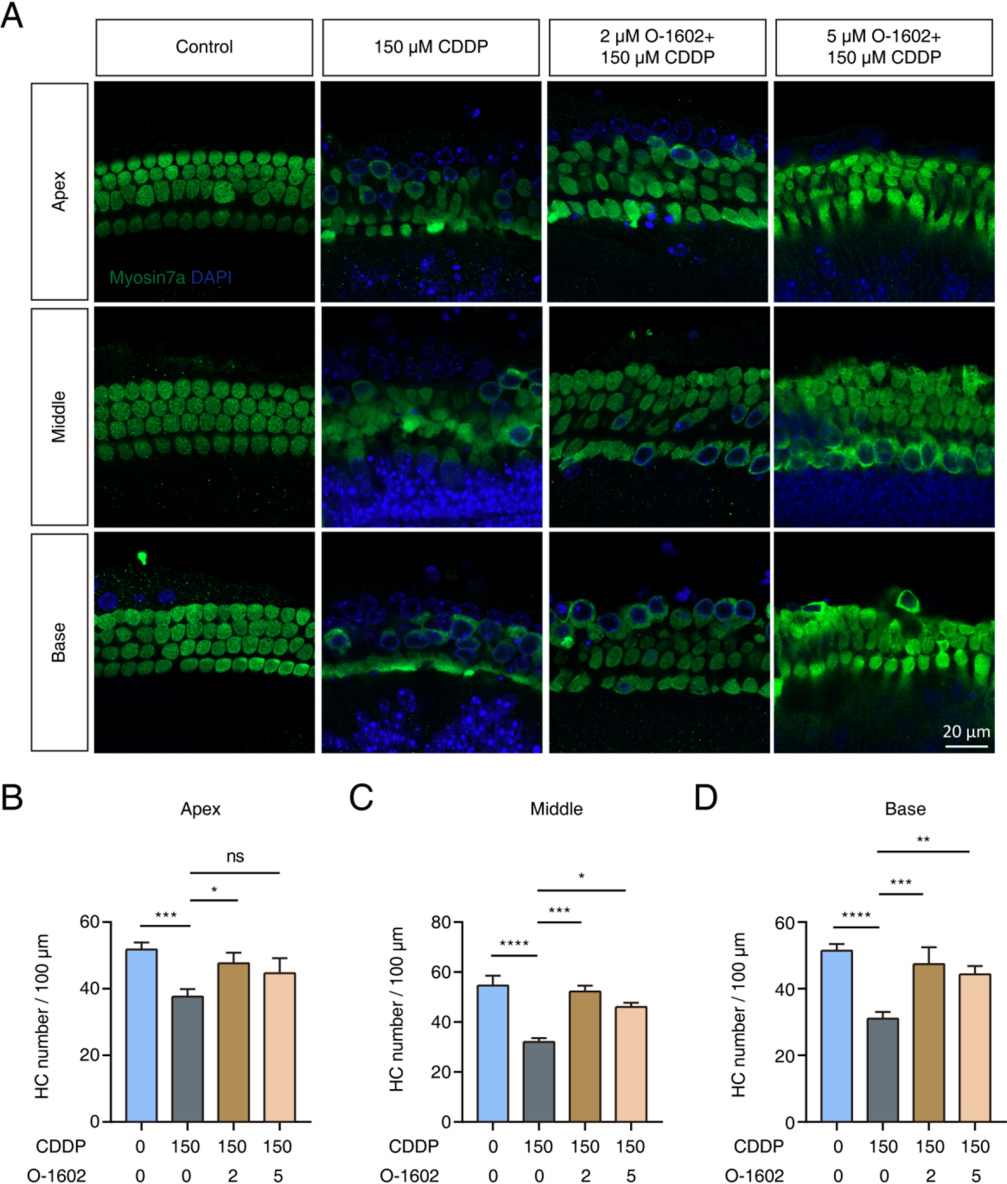
O-1602 improved the survival of HCs after exposure to CDDP. (**A**) Representative images from immunofluorescence staining showed Myosin7a (green) labeling HCs in the apical, middle, and basal turns across four experimental groups. Cochlear explants were pretreated with either 2 or 5 µM O-1602 for 2 h before receiving 150 µM CDDP for 24 h. (**B-D**) Quantification of Myosin7a-positive cell numbers was performed in the apical, middle, and basal turns, respectively. HC: hair cell.

We investigated the effect of GPR55 activation on HCs in cochlear explants. These explants were pretreated with either 2 or 5 µM of O-1602 for 2 h, followed by exposure to 150 µM CDDP for 24 h. Figure 4 presents the survival of HCs under different treatment conditions. The number of HCs in the basal, middle, and apical regions of the cochlea was largely lower after treatment compared to baseline. In contrast, pretreatment with either 2 or 5 µM O-1602 promoted HC survival (Fig. 4A–D). These results suggest that O-1602 may protect HCs by mitigating the harmful effects of CDDP treatment.

We further investigated the protective effects of O-1602 on HCs treated with CDDP. Cochlear explants were pretreated with 2 µM O-1602, with or without 10 µM ML-193, followed by exposure to 150 µM CDDP. The number of HCs in the group treated with O-1602, ML-193, and CDDP was lower compared to the group treated with O-1602 and CDDP. Administering 10 µM ML-193 alone did not cause damage to the HCs (Fig. S4A-D). These results imply that activation of GPR55 by O-1602 is important for protecting HCs from cell death in vitro.

### GPR55 activation attenuated CDDP-induced apoptosis and oxidative stress in HCs within cochlear explants

**Fig. 5:**
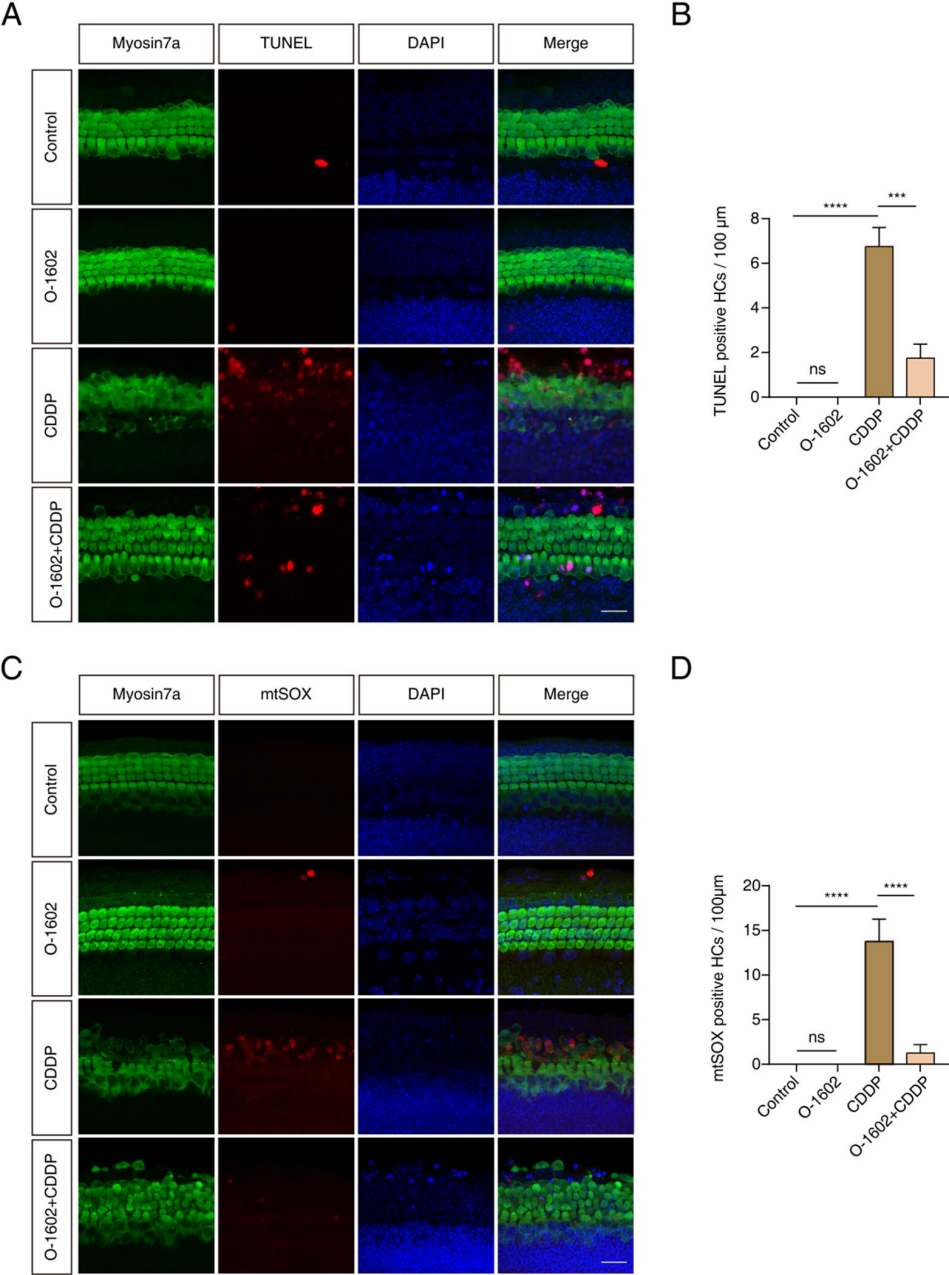
O-1602 diminished the apoptosis and ROS caused by CDDP in HCs within cochlear explants. (**A**,** B**) Representative staining images showed Myosin7a (green) together with TUNEL (red) in the middle turn of the cochlea. (**C**,** D**) Representative staining images showed Myosin7a (green) along with mtSOX (red) in the middle turn of the cochlea. Scale bar = 20 μm. ROS: reactive oxygen species.

We then explored the protective effects of GPR55 on HCs in cultured cochlear explants by performing co-staining with TUNEL and anti-Myosin7a to assess apoptosis levels. Figure 5A and B illustrate that the number of TUNEL-positive HCs was higher following CDDP treatment compared to the control group. In contrast, fewer apoptotic cells were observed in the group pretreated with O-1602. Moreover, administration of O-1602 alone did not considerably increase the levels of apoptosis compared to the control group. These results indicate that GPR55 contributes to reducing CDDP-induced apoptosis in HCs within cochlear explants.

The antioxidative properties of GPR55 were detected in HCs. Cochlear explants were co-stained with mtSOX Deep Red and Myosin7a antibody, and the double-stained cells were counted. The number of mtSOX Deep Red-positive HCs increased following CDDP treatment compared to baseline. However, this increase was less pronounced in samples pretreated with O-1602. Administration of O-1602 alone did not affect oxidative stress levels (Fig. 5C, D). Collectively, these findings suggest that GPR55 alleviates the oxidative stress caused by CDDP in the HCs of cochlear explants.

### GPR55 mitigated CDDP-induced ototoxicity via inhibiting the MAPK pathway

**Fig. 6:**
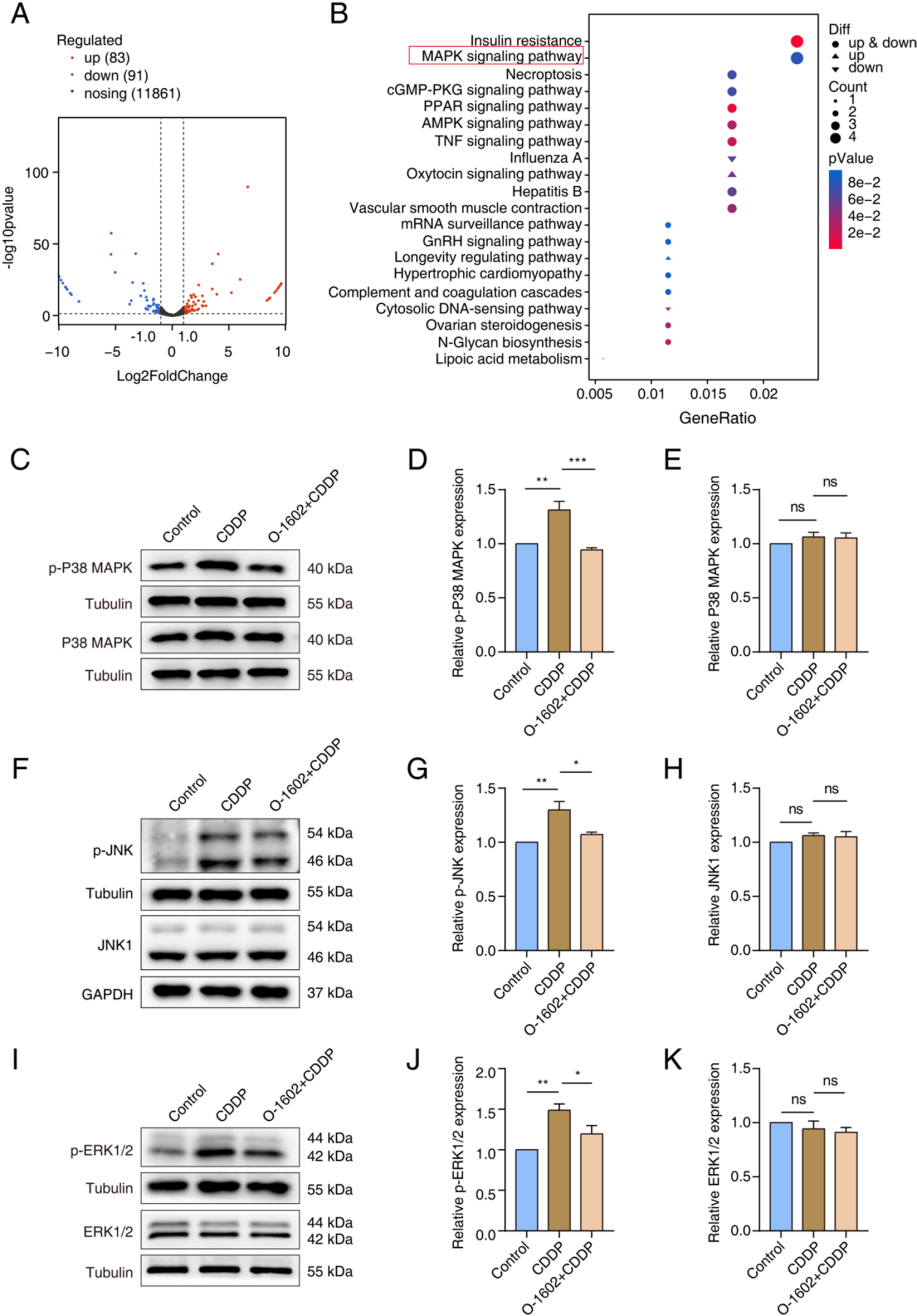
O-1602 protected HCs from damage induced by CDDP via inhibiting the MAPK pathway. (**A**) A volcano plot illustrated the differentially expressed genes between the CDDP group and the O-1602 + CDDP group. (**B**) KEGG analysis displayed the top 20 pathways most significantly affected between the CDDP group and the O-1602 + CDDP group. (**C-E**) Western blot analysis showed the expression levels of p-P38 MAPK and total P38 MAPK among different groups. (**F-H**) Western blot analysis showed the expression levels of p-JNK and total JNK1 among different groups. (**I-K**) Western blot analysis showed the expression levels of p-ERK1/2 and total ERK1/2 among different groups.

We investigated the mechanisms by which O-1602 exerts protective effects using RNA-seq to identify transcriptome-wide alterations between the CDDP group and the O-1602 + CDDP group in HEI-OC1 cells. A total of 174 genes were differentially expressed between the two groups, with 83 genes upregulated and 91 genes downregulated (Fig. 6A; Supplementary Table 2). We assessed the functional implications of these genes through KEGG pathway analysis, which identified the top 20 pathways exhibiting the most significant differences between the groups. Notably, one of the top-ranked pathways was the MAPK pathway, which is strongly associated with oxidative stress and apoptosis (Fig. 6B). We clarified the function of O-1602 in modulating the MAPK signaling cascade by using Western blotting to evaluate the levels of key proteins involved in this pathway. Exposure to CDDP markedly increased the expression levels of phosphorylated P38 MAPK (p-P38), phosphorylated JNK (p-JNK), and phosphorylated ERK1/2 (p-ERK1/2). However, pretreatment with O-1602 reversed these phosphorylation increases. Furthermore, the total protein levels of P38 MAPK, JNK1, and ERK1/2 remained largely unchanged across the groups (Fig. 6C-K). These results suggest that GPR55 protects HCs from CDDP-induced damage primarily by modulating the MAPK signaling pathway.

### Activating GPR55 mitigated CDDP-induced hearing loss in vivo

**Fig. 7:**
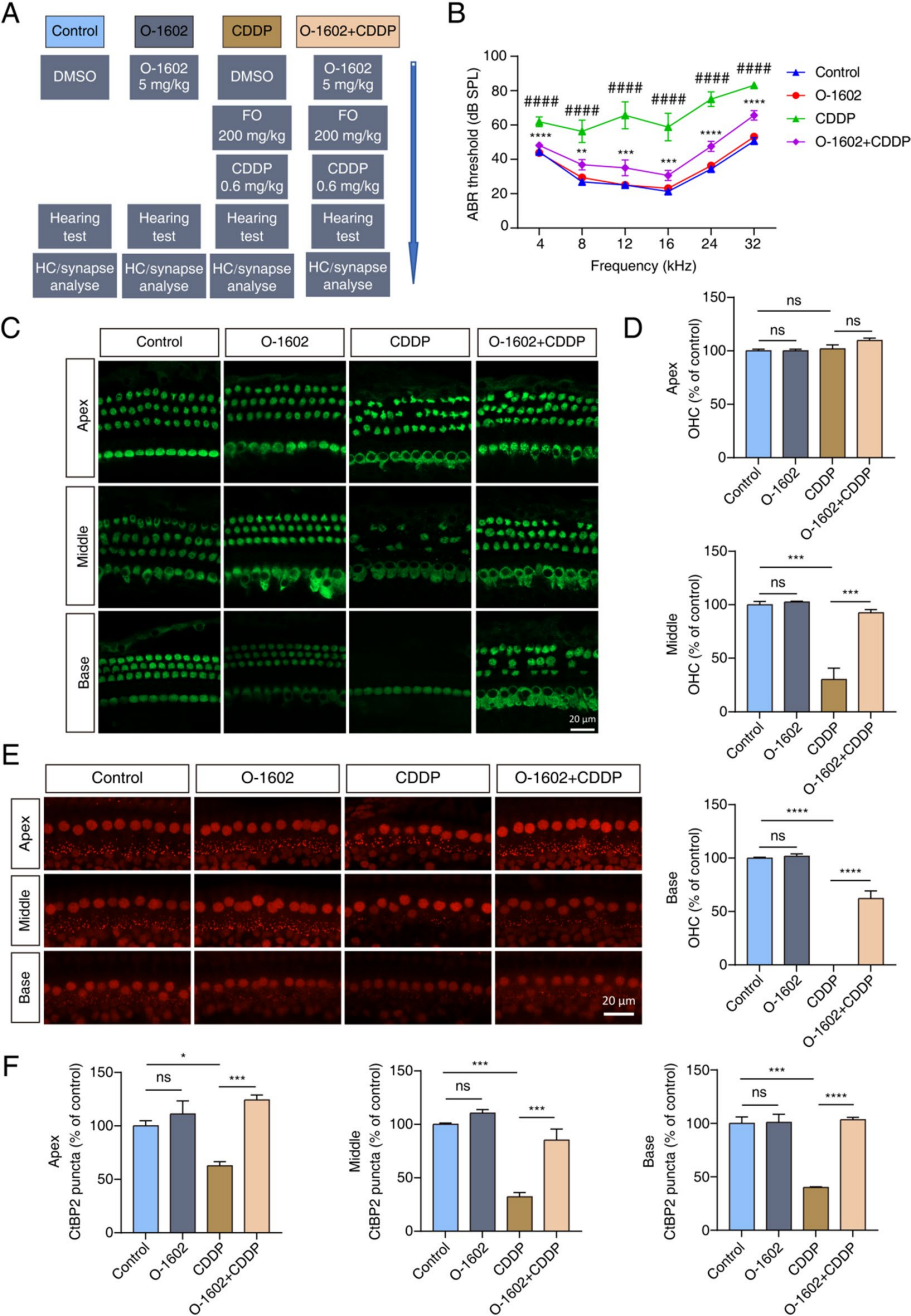
O-1602 mitigated ototoxicity induced by CDDP in an in vivo mouse model. (**A**) A schematic representation showed the workflow for drug administration in mice. FO (200 mg/kg) was given 30 min before the CDDP injection to enhance the ototoxic effects caused by CDDP. The study included four experimental groups: control, O-1602 (5 mg/kg), CDDP (0.6 mg/kg), and O-1602 combined with CDDP. (**B**) ABR thresholds at all frequencies were measured after 7 days of drug administration (*n* = 8 per group). (**C-D**) Immunofluorescence staining images demonstrated Myosin7a-positive (green) HCs in the cochlear region across all experimental groups. (**E**,** F**) Immunofluorescence staining images depicted CtBP2-labeled ribbon synapses (red) in the IHC area. ^#^: Control vs. CDDP; ^*^: CDDP vs. O-1602 + CDDP. FO: furosemide.

Given that the protective effects of GPR55 have been confirmed in vitro, we evaluated its protective effects against hearing impairment in vivo using a mouse model of CDDP-induced ototoxicity. CDDP was administered following established protocols, along with 200 mg/kg of FO to enhance the ototoxic effects (Fig. 7A). We selected 5 mg/kg of O-1602 as the effective dose based on previous research^33^. CDDP treatment led to considerable hearing loss, as evidenced by a marked increase in ABR thresholds at all tested frequencies compared to the control group. However, mice pretreated with O-1602 exhibited lower ABR thresholds than those without pretreatment, suggesting some degree of hearing recovery (Fig. 7B). Cochlear samples were collected for further analysis. Our results revealed considerable outer hair cell (OHC) loss in the middle and basal regions of the cochlea caused by CDDP, while the apical region exhibited minimal damage. Pretreatment with O-1602 increased the number of OHCs in the middle and basal turns (Fig. 7C, D). There was no effect on inner hair cells (IHCs) in any of the groups (Fig. 7C). These findings suggest that GPR55 protects against CDDP-induced hearing loss and HC damage in vivo. Considering the ototoxic properties of FO, a diuretic agent, we administered a single injection of 200 mg/kg of FO. The ABR results showed no significant difference in hearing thresholds between the FO-treated group and the control group (Fig. S5A). Additionally, immunofluorescence analysis demonstrated that the HCs remained intact and well-organized after a single FO injection, suggesting that administering 200 mg/kg of FO alone does not cause ototoxicity in this case (Fig. S5B).

In the cochlea, ribbon synapses establish connections between IHCs and the auditory nerve and are especially susceptible to the ototoxic effects of CDDP^17^. Our study investigating the effects of O-1602 on these synapses demonstrated a significant reduction in ribbon synapse density in the apical, middle, and basal regions of the cochlea in the CDDP-treated group. However, in the group pretreated with O-1602, there was a notable prevention of ribbon synapse degeneration caused by CDDP (Fig. 7E, F). Overall, the study indicates that O-1602 mitigates hearing loss, OHC damage, and ribbon synapse degeneration induced by CDDP in vivo.

## Discussion

CDDP is a potent chemotherapeutic agent widely used to treat solid tumors. However, its application is limited by side effects on normal tissues, including nephrotoxicity, neurotoxicity, and ototoxicity, which negatively affect patients’ quality of life and limit the overall effectiveness of cancer treatment^34–36^. Approximately 40–60% of patients undergoing CDDP therapy experience progressive and irreversible hearing loss, highlighting the severity of this issue^37^. The damaging effects of CDDP are exacerbated by the overaccumulation of ROS, which compromises intracellular defense mechanisms and leads to permanent hearing loss^9,38^. Given the absence of a universally effective treatment to prevent CDDP-induced ototoxicity, we are motivated to explore novel targets for developing such therapies.

Activating certain GPCRs within the cochlea plays a crucial role in protecting against hearing impairment and HC loss^12^. GPR55, a member of the seven-transmembrane GPCR family involved in cannabinoid signaling^39^, and also exhibits antioxidant and anti-inflammatory properties^26^. This study reveals an increase in GPR55 expression levels following CDDP exposure in both HEI-OC1 cells and cultured cochlear explants. Pretreatment with O-1602 preserved the viability of HEI-OC1 cells and the survival of cochlear HCs after CDDP exposure in vitro. Moreover, intraperitoneal administration of O-1602 before CDDP notably reduced ABR thresholds and increased OHC and ribbon synapse survival. Collectively, these findings suggest that GPR55 could be a promising therapeutic target for mitigating the ototoxic effects of CDDP. Considering that another cannabinoid receptor, CB2R, also protects against CDDP-induced hearing loss^17^, we hypothesize that cannabinoid receptors may function as an innate defense system against such damage.

HC apoptosis is the primary mechanism responsible for CDDP-induced ototoxicity^40^. GPR55 has been shown to protect the hippocampus and frontal cortex in Alzheimer’s disease, as well as pancreatic β cells, by inhibiting apoptosis^30,41^. Our results reveal that apoptosis increased in HEI-OC1 cells and HCs following CDDP treatment, consistent with previous studies^42,43^. However, this increase was lessened when HEI-OC1 cells and HCs were pretreated with O-1602. Moreover, O-1602 effectively inhibited apoptosis through both intrinsic and extrinsic pathways, as evidenced by reductions in the mRNA levels of *Caspase-3*, *Apaf-1*, and *FADD*, along with an increase in *Bcl-2* mRNA levels^44^. In cochlear explants in vitro, we observed apoptotic cells that did not co-label with HCs, suggesting the involvement of other cell types, such as supporting cells. This finding aligns with earlier reports that CDDP induces apoptosis in supporting cells^45^. Overall, GPR55 offers protection against CDDP-induced ototoxicity by suppressing apoptotic pathways.

Oxidative stress is linked to HC damage; the overproduction of ROS overwhelms the antioxidative defense system, leading to HC injury and subsequent hearing loss. Therefore, antioxidant compounds such as sodium thiosulfate and N-Acetylcysteine have been used to mitigate the ototoxic effects of CDDP^46,47^. In this study, we expanded research on the antioxidative properties of O-1602 in the context of CDDP-induced ototoxicity. The findings indicate that O-1602 effectively reduces CDDP-induced ROS accumulation and the expression of oxidative stress-related proteins. These findings imply that O-1602 could be a promising candidate for future studies aimed at protecting HCs from oxidative stress-induced damage.

O-1602 is a selective agonist for GPR55, showing strong affinity for this receptor but no affinity for CB1R or CB2R^48^. The ability of O-1602 to activate GPR55 has prompted studies exploring this pathway; however, other GPCRs may also act as alternative targets for O-1602^49,50^. To pinpoint the specific mechanisms underlying O-1602’s effects, we used the selective antagonist ML-193 to inhibit GPR55 activation. Our findings indicate that the protective effects of O-1602 on HCs and HEI-OC1 cells were abolished when ML-193 and O-1602 were administered simultaneously. This finding supports the conclusion that O-1602’s protective role in HCs depends on GPR55.

The MAPK pathway is essential in the eukaryotic signaling network by regulating cellular processes such as differentiation, proliferation, and apoptosis^51,52^. This pathway comprises several key components, including P38 MAPK, JNK, ERK1/2, and ERK5, each activated by different stimuli^52,53^. MAPK signaling has been reported to be associated with hearing loss^54^. Multiple studies indicate that activation of the MAPK signaling pathway, particularly P38 MAPK, JNK, and ERK1/2, may be a primary mechanism underlying CDDP-induced hearing damage^45,55,56^. Consequently, blocking the activation of these pathways may help prevent HC death caused by CDDP^57,58^. In this study, the P38 MAPK, JNK, and ERK1/2 pathways were activated in response to CDDP exposure. Pretreatment with O-1602 reversed the elevated levels of these proteins, indicating that GPR55 protects against CDDP-induced ototoxicity via suppressing the MAPK pathway. The suppressive effect of GPR55 on the MAPK pathway is consistent with its identity as a GPCR. Other GPCR family members, such as DRD4, have been demonstrated to negatively regulate MAPK signaling through various mechanisms, thereby mediating cytoprotective effects^59^. Interestingly, in certain models, such as tumor research, reducing GPR55 levels results in decreased MAPK activation^60,61^. This study highlights the complexity and context-specific characteristics of GPCR signaling. These differences may arise from variations in receptor environments, the distinct properties of interacting proteins, or the diversity of stimuli involved. In the case of CDDP-induced ototoxicity, intense apoptotic stress may selectively activate specific signaling complexes, leading GPR55 to play a protective role by suppressing MAPK activation. Future research will focus on identifying the precise downstream partners involved. In summary, these findings enhance our understanding of GPR55’s function and provide novel strategies for targeting GPCR-MAPK pathways to prevent CDDP-induced hearing loss.

## Conclusions

**Fig. 8:**
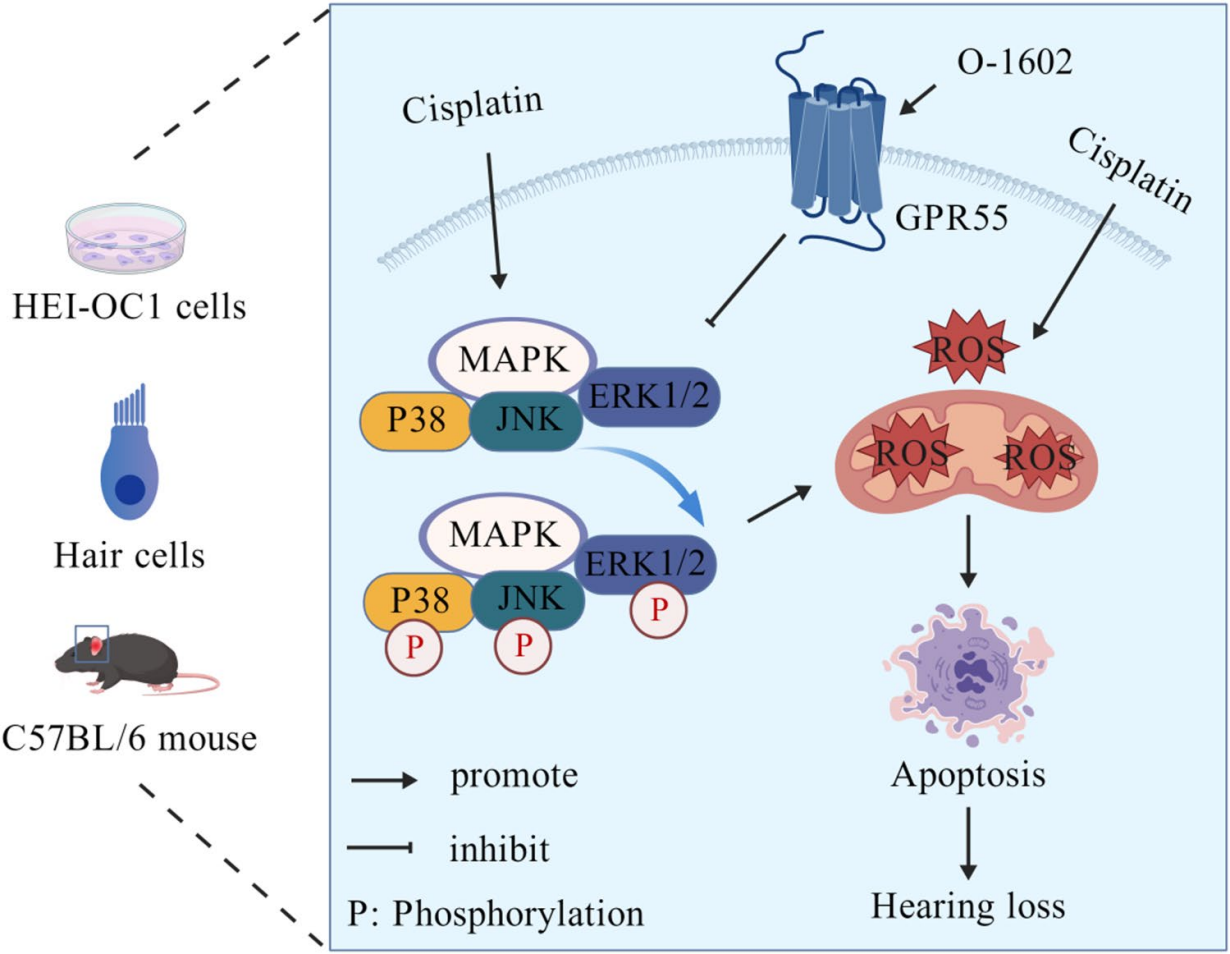
Schematic of GPR55 function in preventing hearing damage caused by CDDP. Activation of GPR55 by O-1602 protected against CDDP-induced ototoxicity in HEI-OC1 cells, HCs within cochlear explants, and in vivo mouse models by inhibiting the MAPK pathway.

The role of GPR55 in the auditory system was examined. Our results showed that GPR55 is present in the cochlea, with significantly increased expression in cochlear explants and HEI-OC1 cells following CDDP exposure. Furthermore, activation of GPR55 by O-1602 increased HC survival and HEI-OC1 cell viability in vitro, and mitigated CDDP-induced hearing loss, OHC loss, and ribbon synapse degeneration in a mouse model in vivo, indicating the protective effects of O-1602 on the auditory system. In addition, O-1602 suppressed apoptosis and reduced ROS accumulation, primarily through inhibiting the MAPK pathway (as illustrated in Fig. 8, created with BioGDP.com)^62^. These findings suggest that GPR55 is a promising therapeutic target for combating the ototoxic effects caused by CDDP exposure.

## Supplementary Information

Below is the link to the electronic supplementary material.

Supplementary Material 1

Supplementary Material 2

Supplementary Material 3

Supplementary Material 4

## Data Availability

The authors declare that the data supporting the findings of this study are available within the paper and its Supplementary Information files. The sequence data in this study are obtained from the NCBI Sequence Read Archive (SRA) under the accession numbers PRJNA1426777.

## References

[1] Nieman, C. L. & Oh, E. S. Hearing Loss. *Ann. Intern. Med.* **173**, C81–C96 (2020). 10.7326/AITC202012010 33253610

[2] Vlajkovic, S. M. & Thorne, P. R. Molecular mechanisms of sensorineural hearing loss and development of inner ear therapeutics. *Int. J. Mol. Sci. ***22**, 5647 10.3390/ijms22115647 (2021). 34073285 10.3390/ijms22115647 PMC8198622

[3] Lee, J., Fernandez, K. & Cunningham, L. L. Hear and now: Ongoing clinical trials to prevent drug-induced hearing loss. *Annu. Rev. Pharmacol. Toxicol.* **64**, 211–230 (2024). 37562496 10.1146/annurev-pharmtox-033123-114106

[4] Fischer, N., Weber, B. & Riechelmann, H. [Presbycusis - Age Related Hearing Loss]. *Laryngorhinootologie* **95**, 497–510 (2016). 27392191 10.1055/s-0042-106918

[5] Romano, A. et al. Platinum-induced ototoxicity and hearing impairment in children and adolescents. *Drugs Context.* **14**, 2025–3–1 (2025). 10.7573/dic.2025-3-1 PMC12169122 40524783

[6] Moke, D. J. et al. Prevalence and risk factors for cisplatin-induced hearing loss in children, adolescents, and young adults: A multi-institutional North American cohort study. *Lancet Child Adolesc. Health* **5**, 274–283 (2021). 33581749 10.1016/S2352-4642(21)00020-1 PMC9059427

[7] Wang, X. et al. Cisplatin-induced ototoxicity: From signaling network to therapeutic targets. *Biomed. Pharmacother.* **157**, 114045 (2023). 36455457 10.1016/j.biopha.2022.114045

[8] Maniaci, A. et al. Hearing loss and oxidative stress: A comprehensive review. *Antioxidants (Basel). ***13**, 842 10.3390/antiox13070842 (2024). 39061910 10.3390/antiox13070842 PMC11274311

[9] Ramkumar, V., Mukherjea, D., Dhukhwa, A. & Rybak, L. P. Oxidative stress and inflammation caused by cisplatin ototoxicity. *Antioxidants. ***10**, 1919 10.3390/antiox10121919 (2021). 34943021 10.3390/antiox10121919 PMC8750101

[10] Sheth, S., Mukherjea, D., Rybak, L. P. & Ramkumar, V. Mechanisms of Cisplatin-Induced Ototoxicity and Otoprotection. *Front. Cell. Neurosci.* **11**, 338 (2017). 29163050 10.3389/fncel.2017.00338 PMC5663723

[11] Lorente, J. S. et al. GPCR drug discovery: new agents, targets and indications. *Nat Rev. Drug Discov. ***24**, 458–479 (2025). 10.1038/s41573-025-01139-y 40033110

[12] Zhang, Z. & Chai, R. Hear the sounds: The role of G protein-coupled receptors in the cochlea. *Am. J. Physiol. Cell Physiol.* **323**, C1088–C1099 (2022). 35938679 10.1152/ajpcell.00453.2021

[13] Ma, X. et al. G protein-coupled receptors in cochlea: Potential therapeutic targets for hearing loss. *Front. Mol. Neurosci.* **15**, 1028125 (2022). 36311029 10.3389/fnmol.2022.1028125 PMC9596917

[14] Kaur, T. et al. Adenosine A1 receptor protects against cisplatin ototoxicity by suppressing the NOX3/STAT1 inflammatory pathway in the cochlea. *J. Neurosci.* **36**, 3962–3977 (2016). 27053204 10.1523/JNEUROSCI.3111-15.2016 PMC4821909

[15] Vlajkovic, S. M. et al. Adenosine receptors regulate susceptibility to noise-induced neural injury in the mouse cochlea and hearing loss. *Hear. Res.* **345**, 43–51 (2017). 28034618 10.1016/j.heares.2016.12.015

[16] Vlajkovic, S. M. et al. Adenosine kinase inhibition in the cochlea delays the onset of age-related hearing loss. *Exp. Gerontol.* **46**, 905–914 (2011). 21846498 10.1016/j.exger.2011.08.001 PMC3200489

[17] Ghosh, S. et al. The endocannabinoid/cannabinoid receptor 2 system protects against cisplatin-induced hearing loss. *Front. Cell. Neurosci.* **12**, 271 (2018). 30186120 10.3389/fncel.2018.00271 PMC6110918

[18] Martin-Saldana, S. et al. Spontaneous cannabinoid receptor 2 (CB2) expression in the cochlea of adult albino rat and its up-regulation after cisplatin treatment. *PLoS One* **11**, e161954 (2016). 10.1371/journal.pone.0161954 PMC5001640 27564061

[19] Jeong, H. J. et al. Antiapoptotic mechanism of cannabinoid receptor 2 agonist on cisplatin-induced apoptosis in the HEI-OC1 auditory cell line. *J. Neurosci. Res.* **85**, 896–905 (2007). 17183590 10.1002/jnr.21168

[20] Pertwee, R. G. GPR55: A new member of the cannabinoid receptor clan?. *Br. J. Pharmacol.* **152**, 984–986 (2007). 17876300 10.1038/sj.bjp.0707464 PMC2095104

[21] Ryberg, E. et al. The orphan receptor GPR55 is a novel cannabinoid receptor. *Br. J. Pharmacol.* **152**, 1092–1101 (2007). 17876302 10.1038/sj.bjp.0707460 PMC2095107

[22] Peng, J. et al. A narrative review of molecular mechanism and therapeutic effect of cannabidiol (CBD). *Basic Clin. Pharmacol. Toxicol.* **130**, 439–456 (2022). 35083862 10.1111/bcpt.13710

[23] Calvillo-Robledo, A., Cervantes-Villagrana, R. D., Morales, P. & Marichal-Cancino, B. A. The oncogenic lysophosphatidylinositol (LPI)/GPR55 signaling. *Life Sci.* **301**, 120596 (2022). 35500681 10.1016/j.lfs.2022.120596

[24] Alhouayek, M., Masquelier, J. & Muccioli, G. G. Lysophosphatidylinositols, from cell membrane constituents to GPR55 ligands. *Trends Pharmacol. Sci.* **39**, 586–604 (2018). 29588059 10.1016/j.tips.2018.02.011

[25] Harada, N. et al. Identification of G protein-coupled receptor 55 (GPR55) as a target of curcumin. *NPJ Sci. Food* **6**, 4 (2022). 35031622 10.1038/s41538-021-00119-x PMC8760322

[26] Liu, B., Song, S., Jones, P. M. & Persaud, S. J. GPR55: From orphan to metabolic regulator?. *Pharmacol. Ther.* **145**, 35–42 (2015). 24972076 10.1016/j.pharmthera.2014.06.007

[27] Shi, Q. X. et al. The novel cannabinoid receptor GPR55 mediates anxiolytic-like effects in the medial orbital cortex of mice with acute stress. *Mol. Brain* **10**, 38 (2017). 28800762 10.1186/s13041-017-0318-7 PMC5553743

[28] Kallendrusch, S. The G protein-coupled receptor 55 ligand l-alpha-lysophosphatidylinositol exerts microglia-dependent neuroprotection after excitotoxic lesion. *Glia* **61**, 1822–1831 (2013). 24038453 10.1002/glia.22560

[29] Minamihata, T., Takano, K., Moriyama, M. & Nakamura, Y. Lysophosphatidylinositol, an endogenous ligand for G protein-coupled receptor 55, has anti-inflammatory effects in cultured microglia. *Inflammation* **43**, 1971–1987 (2020). 32519268 10.1007/s10753-020-01271-4

[30] Xiang, X. Activation of GPR55 attenuates cognitive impairment and neurotoxicity in a mouse model of Alzheimer’s disease induced by Abeta(1–42) through inhibiting RhoA/ROCK2 pathway. *Prog. Neuropsychopharmacol. Biol. Psychiatry* **112**, 110423 (2022). 34363866 10.1016/j.pnpbp.2021.110423

[31] Fu, X. et al. Tuberous sclerosis complex-mediated mTORC1 overactivation promotes age-related hearing loss. *J. Clin. Invest.* **128**, 4938–4955 (2018). 30247156 10.1172/JCI98058 PMC6205401

[32] Kotsikorou, E. et al. Identification of the GPR55 antagonist binding site using a novel set of high-potency GPR55 selective ligands. *Biochemistry* **52**, 9456–9469 (2013). 24274581 10.1021/bi4008885 PMC3970910

[33] Schicho, R. et al. The atypical cannabinoid O-1602 protects against experimental colitis and inhibits neutrophil recruitment. *Inflamm. Bowel Dis.* **17**, 1651–1664 (2011). 21744421 10.1002/ibd.21538 PMC3116968

[34] Wellenberg, A. et al. Cisplatin-induced neurotoxicity involves the disruption of serotonergic neurotransmission. *Pharmacol. Res.* **174**, 105921 (2021). 34601079 10.1016/j.phrs.2021.105921

[35] Tang, C., Livingston, M. J., Safirstein, R. & Dong, Z. Cisplatin nephrotoxicity: New insights and therapeutic implications. *Nat. Rev. Nephrol.* **19**, 53–72 (2023). 36229672 10.1038/s41581-022-00631-7

[36] Hodge, S. E., Lopez, I. A., Ishiyama, G. & Ishiyama, A. Cisplatin ototoxicity histopathology. *Laryngoscope Investig. Otolaryngol.* **6**, 852–856 (2021). 34401512 10.1002/lio2.608 PMC8356863

[37] Marshak, T., Steiner, M., Kaminer, M., Levy, L. & Shupak, A. Prevention of cisplatin-induced hearing loss by intratympanic dexamethasone: A randomized controlled study. *Otolaryngol. Head Neck Surg.* **150**, 983–990 (2014). 24618499 10.1177/0194599814524894

[38] Callejo, A., Sedo-Cabezon, L., Juan, I. D. & Llorens, J. Cisplatin-induced ototoxicity: Effects, mechanisms and protection strategies. *Toxics* **3**, 268–293 (2015). 29051464 10.3390/toxics3030268 PMC5606684

[39] Sharir, H. & Abood, M. E. Pharmacological characterization of GPR55, a putative cannabinoid receptor. *Pharmacol. Ther.* **126**, 301–313 (2010). 20298715 10.1016/j.pharmthera.2010.02.004 PMC2874616

[40] Wu, P. et al. Hair cell protection from ototoxic drugs. *Neural Plast.* **2021**, 4909237 (2021). 34335732 10.1155/2021/4909237 PMC8289577

[41] Vong, C. T., Tseng, H., Kwan, Y. W., Lee, S. M. & Hoi, M. Novel protective effect of O-1602 and abnormal cannabidiol, GPR55 agonists, on ER stress-induced apoptosis in pancreatic beta-cells. *Biomed. Pharmacother.* **111**, 1176–1186 (2019). 30841431 10.1016/j.biopha.2018.12.126

[42] He, Y. et al. Inhibiting DNA methylation alleviates cisplatin-induced hearing loss by decreasing oxidative stress-induced mitochondria-dependent apoptosis via the LRP1-PI3K/AKT pathway. *Acta Pharm. Sin B*. **12**, 1305–1321 (2022). 35530135 10.1016/j.apsb.2021.11.002 PMC9069410

[43] Nan, B. et al. Astaxanthine attenuates cisplatin ototoxicity *in vitro* and protects against cisplatin-induced hearing loss *in vivo*. *Acta Pharm. Sin. B* **12**, 167–181 (2022). 35127378 10.1016/j.apsb.2021.07.002 PMC8800030

[44] Kashyap, D., Garg, V. K. & Goel, N. Intrinsic and extrinsic pathways of apoptosis: Role in cancer development and prognosis. *Adv. Protein Chem. Struct. Biol.* **125**, 73–120 (2021). 33931145 10.1016/bs.apcsb.2021.01.003

[45] Nicholas, B. D., Francis, S., Wagner, E. L., Zhang, S. & Shin, J. Protein synthesis inhibition and activation of the c-Jun N-terminal kinase are potential contributors to cisplatin ototoxicity. *Front. Cell. Neurosci.* **11**, 303 (2017). 29033791 10.3389/fncel.2017.00303 PMC5627031

[46] Rybak, L. P., Mukherjea, D., Jajoo, S. & Ramkumar, V. Cisplatin ototoxicity and protection: Clinical and experimental studies. *Tohoku J. Exp. Med.* **219**, 177–186 (2009). 19851045 10.1620/tjem.219.177 PMC2927105

[47] Rybak, L. P., Mukherjea, D. & Ramkumar, V. Mechanisms of cisplatin-induced ototoxicity and prevention. *Semin. Hear.* **40**, 197–204 (2019). 31036996 10.1055/s-0039-1684048 PMC6486366

[48] Ashton, J. C. The atypical cannabinoid O-1602: Targets, actions, and the central nervous system. *Cent. Nerv. Syst. Agents Med. Chem.* **12**, 233–239 (2012). 22831390 10.2174/187152412802430156

[49] McHugh, D. et al. N-arachidonoyl glycine, an abundant endogenous lipid, potently drives directed cellular migration through GPR18, the putative abnormal cannabidiol receptor. *BMC Neurosci.* **11**, 44 (2010). 20346144 10.1186/1471-2202-11-44 PMC2865488

[50] Diaz-Arteaga, A. et al. The atypical cannabinoid O-1602 stimulates food intake and adiposity in rats. *Diabetes Obes. Metab.* **14**, 234–243 (2012). 21981246 10.1111/j.1463-1326.2011.01515.x

[51] Lin, F. X., Gu, H. Y. & He, W. MAPK signaling pathway in spinal cord injury: Mechanisms and therapeutic potential. *Exp. Neurol.* **383**, 115043 (2025). 39522804 10.1016/j.expneurol.2024.115043

[52] Kim, E. K. & Choi, E. J. Pathological roles of MAPK signaling pathways in human diseases. *Biochim. Biophys. Acta* **1802**, 396–405 (2010). 20079433 10.1016/j.bbadis.2009.12.009

[53] McCubrey, J. A., Lahair, M. M. & Franklin, R. A. Reactive oxygen species-induced activation of the MAP kinase signaling pathways. *Antioxid. Redox Signal.* **8**, 1775–1789 (2006). 16987031 10.1089/ars.2006.8.1775

[54] Liu, Y. et al. Key signaling pathways regulate the development and survival of auditory hair cells. *Neural Plast.* **2021**, 5522717 (2021). 34194486 10.1155/2021/5522717 PMC8214483

[55] So, H. et al. Evidence that cisplatin-induced auditory damage is attenuated by downregulation of pro-inflammatory cytokines via Nrf2/HO-1. *J. Assoc. Res. Otolaryngol.* **9**, 290–306 (2008). 18584244 10.1007/s10162-008-0126-y PMC2538144

[56] Li, Y. et al. 3-hydroxy-3-methylglutaryl-coenzyme A (HMG-CoA) reductase (HMGCR) protects hair cells from cisplatin-induced ototoxicity *in vitro*: Possible relation to the activities of p38 MAPK signaling pathway. *Arch. Toxicol.* **97**, 2955–2967 (2023). 37608195 10.1007/s00204-023-03588-z

[57] Wang, J. et al. A peptide inhibitor of c-Jun N-terminal kinase protects against both aminoglycoside and acoustic trauma-induced auditory hair cell death and hearing loss. *J. Neurosci.* **23**, 8596–8607 (2003). 13679429 10.1523/JNEUROSCI.23-24-08596.2003 PMC6740364

[58] Wei, X. et al. Minocycline prevents gentamicin-induced ototoxicity by inhibiting p38 MAP kinase phosphorylation and caspase 3 activation. *Neuroscience* **131**, 513–521 (2005). 15708492 10.1016/j.neuroscience.2004.11.014

[59] Gao, Y. et al. DRD4 alleviates acute kidney injury by suppressing ISG15/NOX4 axis-associated oxidative stress. *Redox biology* **70**, 103078 (2024). 38354631 10.1016/j.redox.2024.103078 PMC10876914

[60] Ferro, R. et al. GPR55 signalling promotes proliferation of pancreatic cancer cells and tumour growth in mice, and its inhibition increases effects of gemcitabine. *Oncogene* **37**, 6368–6382 (2018). 30061636 10.1038/s41388-018-0390-1

[61] Tan, S., Zaman, Q. U., Fahad, S. & Deng, G. Cannabidiol reverts the malignant phenotype of hepatocellular carcinoma cells via the GPR55/TP53/MAPK axis. *Biochimica et Biophysica Acta (BBA)* **1868**, 130651 (2024). 10.1016/j.bbagen.2024.130651 38825256

[62] Jiang, S. et al. Generic diagramming platform (GDP): A comprehensive database of high-quality biomedical graphics. *Nucleic Acids Res.* **53**, D1670–D1676 (2025). 39470721 10.1093/nar/gkae973 PMC11701665

