# Supplementary material for: Activating GPR55 protects cochlear hair cells against cisplatin-induced ototoxicity via inhibiting MAPK pathway
Source: Sci Rep. 2026 Apr 14;16:17287. doi: 10.1038/s41598-026-48548-6 (PMC13234428; doi:10.1038/s41598-026-48548-6)
Supplement: Supplementary file 1 — Supplementary Material 1 [file 41598_2026_48548_MOESM1_ESM.pdf]

Fig. 1C

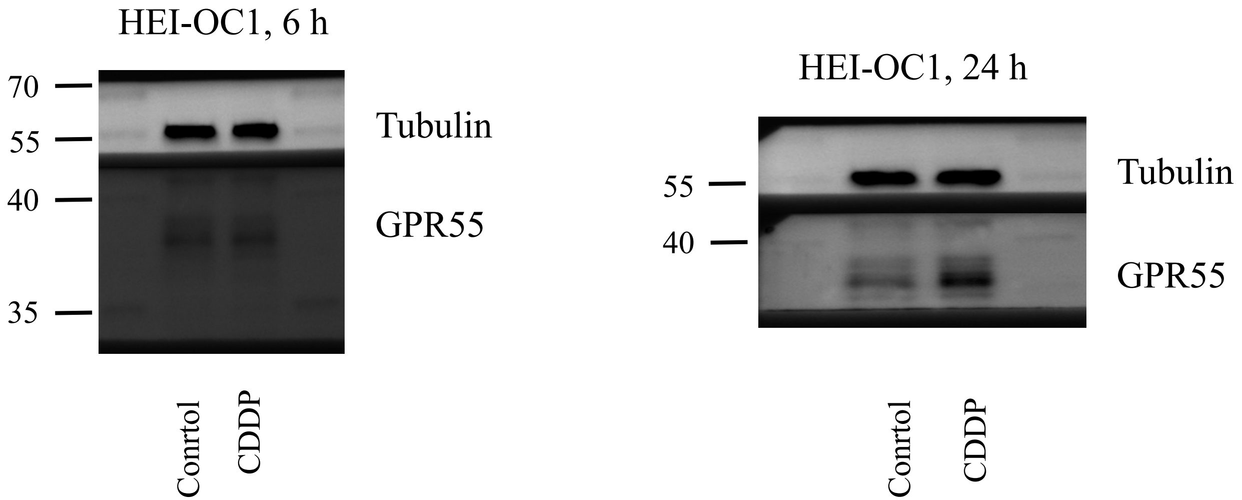

Fig. 1C shows the whole blot after cutting membrane at molecular weight 35~40 kDa for GPR55 (37 kDa), and 55~70 kDa for Tubulin (55 kDa).

Fig. 1G

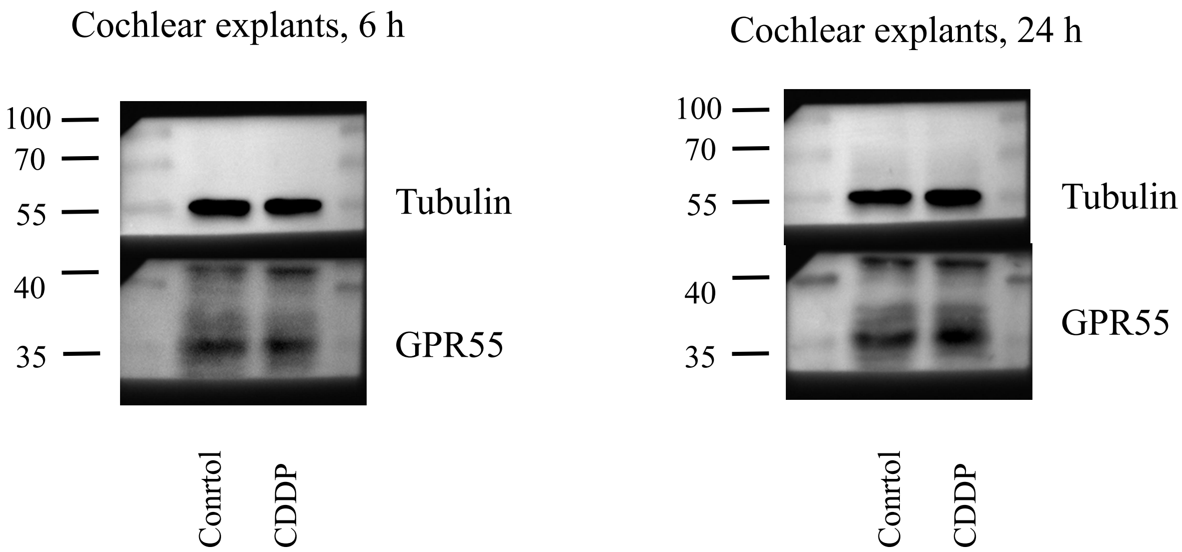

Fig. 1G shows the whole blot after cutting membrane at molecular weight 35~40 kDa for GPR55 (37 kDa), and 55~100 kDa for Tubulin (55 kDa).

Fig. 2G

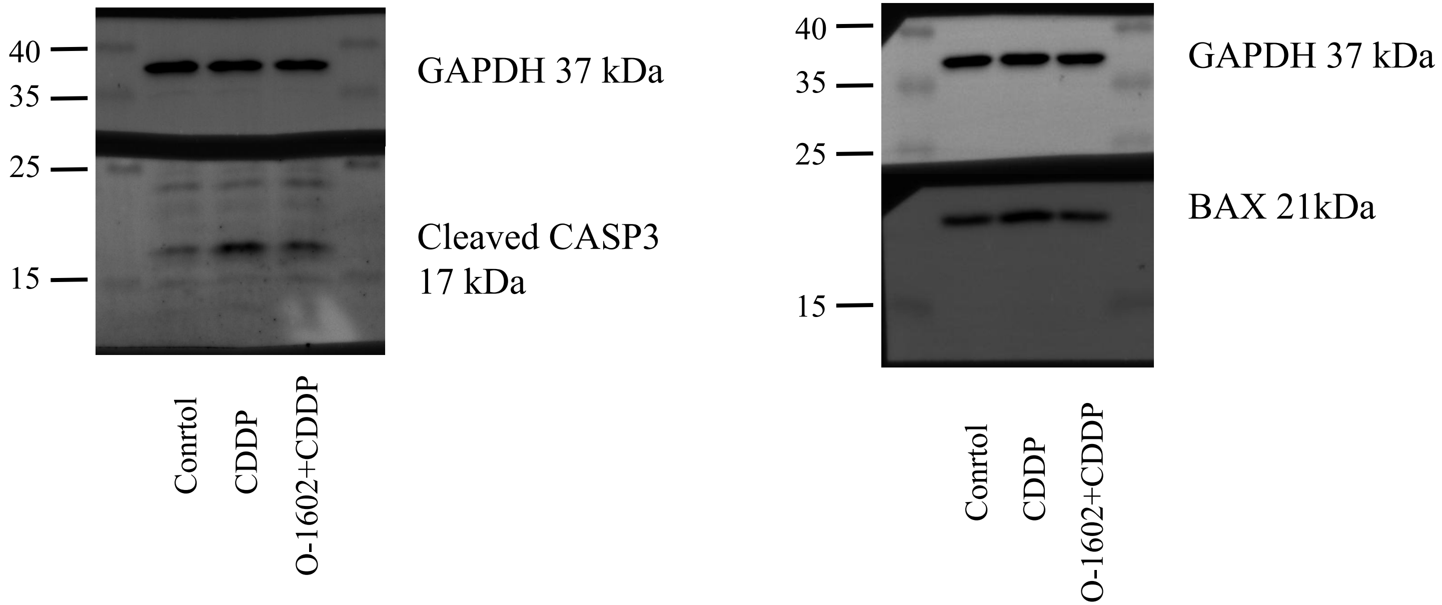

Fig. 2G (left) shows the whole blot after cutting membrane at molecular weight 15~25 kDa for Cleaved CASP3 (17 kDa), and 35~40 kDa for GAPDH (37 kDa).
Fig. 2G (right) shows the whole blot after cutting membrane at molecular weight 15~25 kDa for BAX (21 kDa), and 25~40 kDa for GAPDH (37 kDa).

Fig. 3C

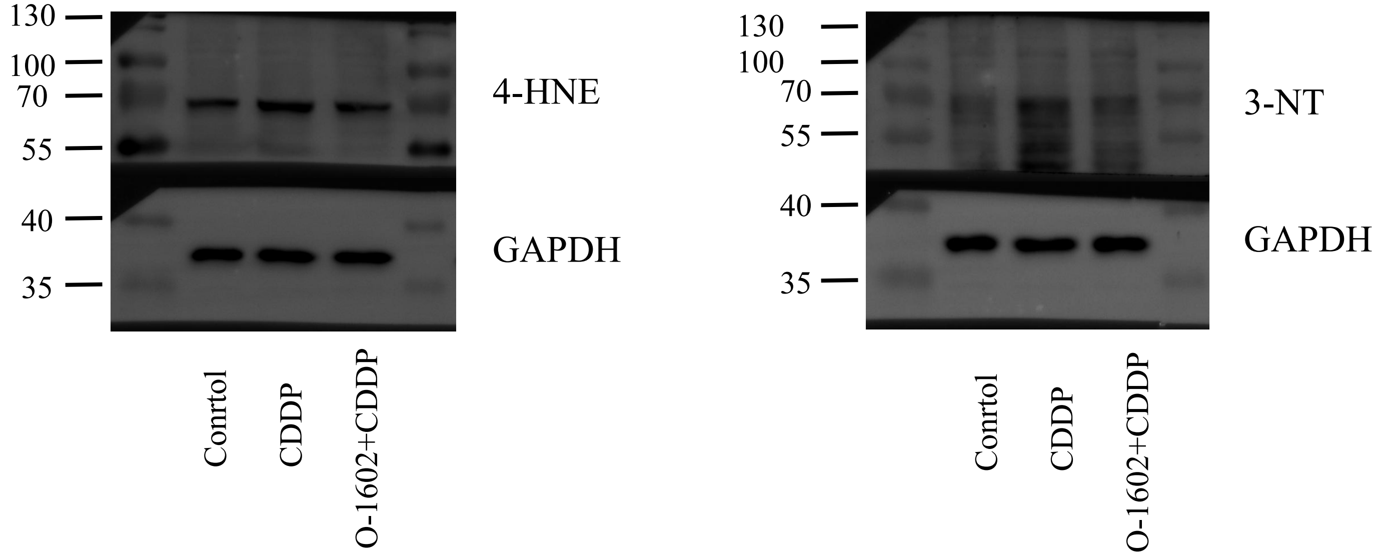

Fig. 3C (left) shows the whole blot after cutting membrane at molecular weight 55~130 kDa for 4-HNE (66 kDa), and 35~40 kDa for GAPDH (37 kDa).
Fig. 3C (right) shows the whole blot after cutting membrane at molecular weight 55~130 kDa for 3-NT (55~70 kDa), and 35~40 kDa for GAPDH (37 kDa).

Fig. 6C

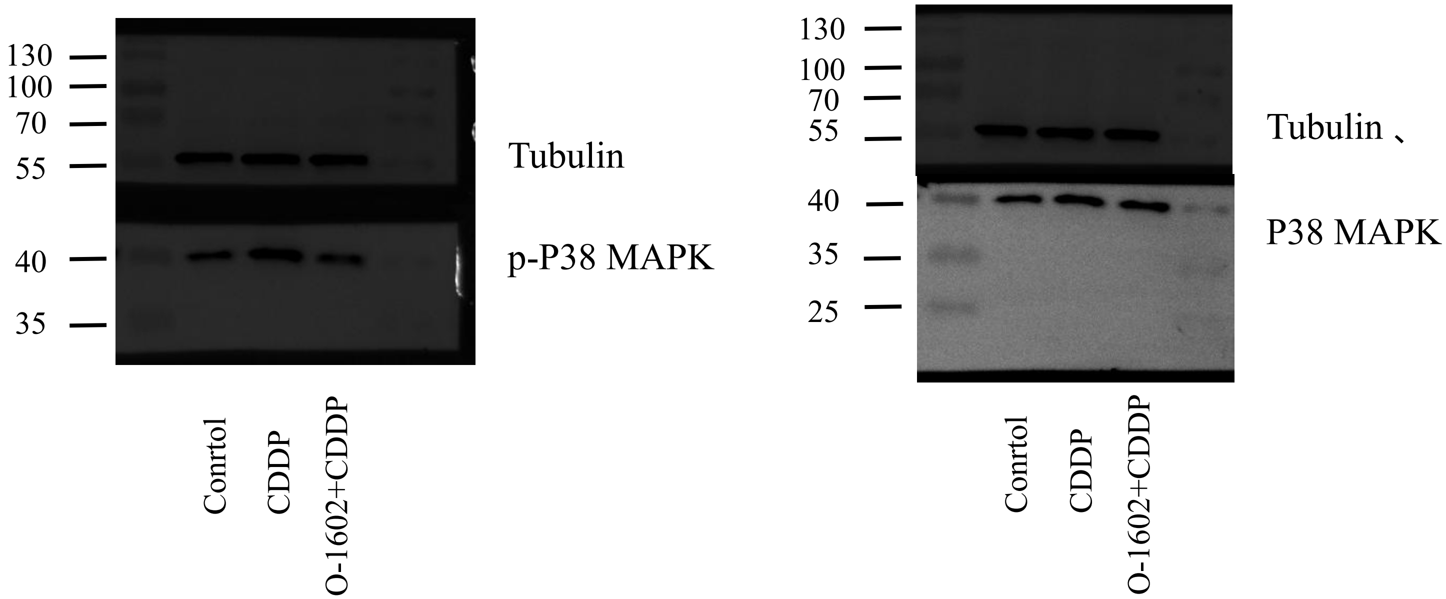

Fig. 6C (left) shows the whole blot after cutting membrane at molecular weight 55~130 kDa for Tubulin (55 kDa), and 35~40 kDa for p-P38 MAPK (40 kDa).
Fig. 6C (right) shows the whole blot after cutting membrane at molecular weight 55~130 kDa for Tubulin (55 kDa), and 25~40 kDa for P38 MAPK (40 kDa).

Fig. 6F

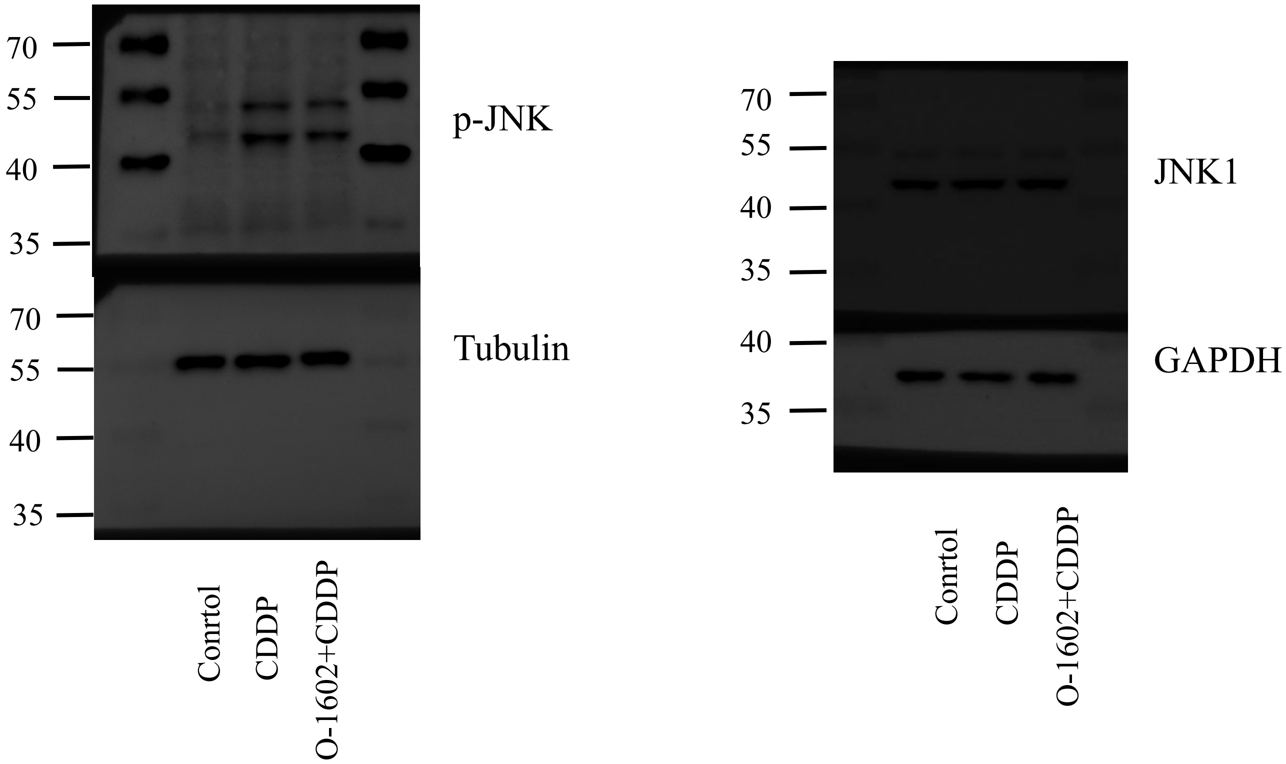

Fig. 6F (left) shows the whole blot after cutting membrane at molecular weight 35~70 kDa for Tubulin (55 kDa), and 35~70 kDa for p-JNK (54, 46 kDa).
Fig. 6F (right) shows the whole blot after cutting membrane at molecular weight 35~40 kDa for GAPDH , and 35~70 kDa for JNK1 (54, 46 kDa).

Fig. 6I

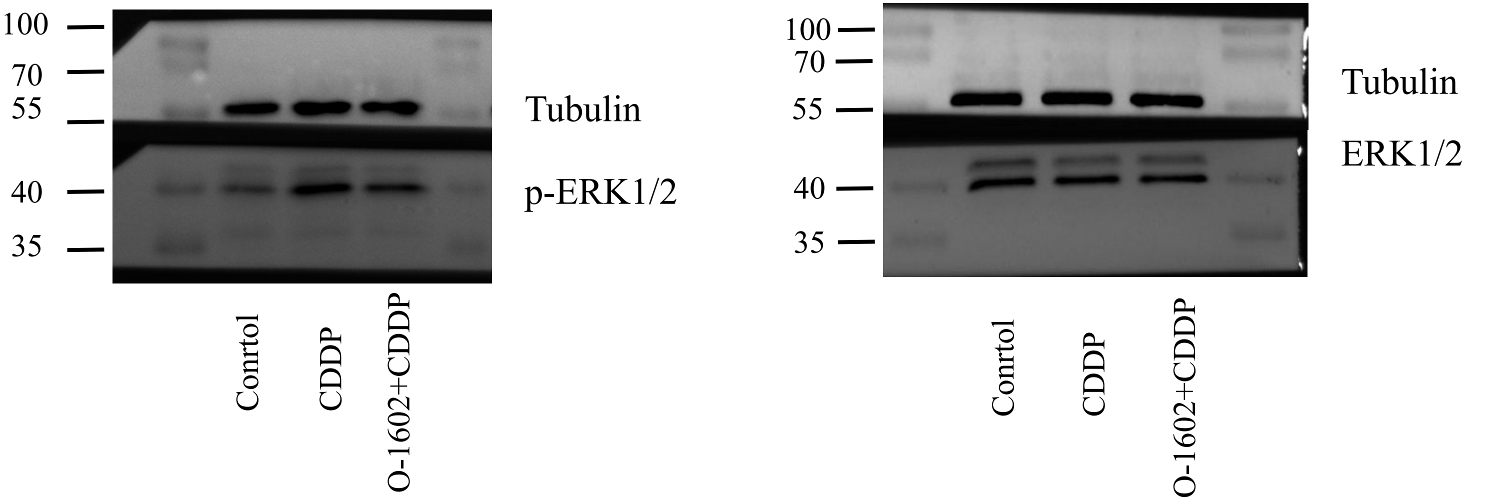

Fig. 6I (left) shows the whole blot after cutting membrane at molecular weight 55~100 kDa for Tubulin (55 kDa), and 35~40+ kDa for p-ERK1/2 (44, 42 kDa).
Fig. 6I (right) shows the whole blot after cutting membrane at molecular weight 55~100 kDa for Tubulin (55 kDa), and 35~40+ kDa for ERK1/2 (44, 42 kDa).

Fig. S3B

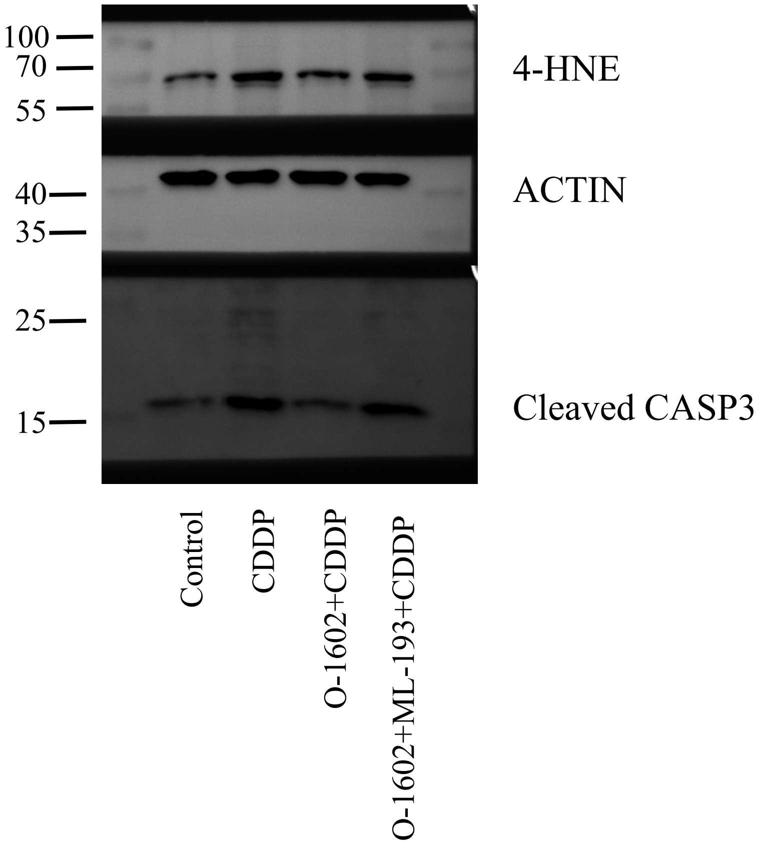

Fig. S3B shows the whole blot after cutting membrane at molecular weight 15~25 kDa for Cleaved CASP3 (17 kDa), 35~40 kDa for ACTIN (42 kDa), and 55~100 kDa for 4-HNE (66 kDa).
