# Supplementary material for: Activating GPR55 protects cochlear hair cells against cisplatin-induced ototoxicity via inhibiting MAPK pathway
Source: Sci Rep. 2026 Apr 14;16:17287. doi: 10.1038/s41598-026-48548-6 (PMC13234428; doi:10.1038/s41598-026-48548-6)
Supplement: Supplementary file 2 — Supplementary Material 2 [file 41598_2026_48548_MOESM2_ESM.docx]

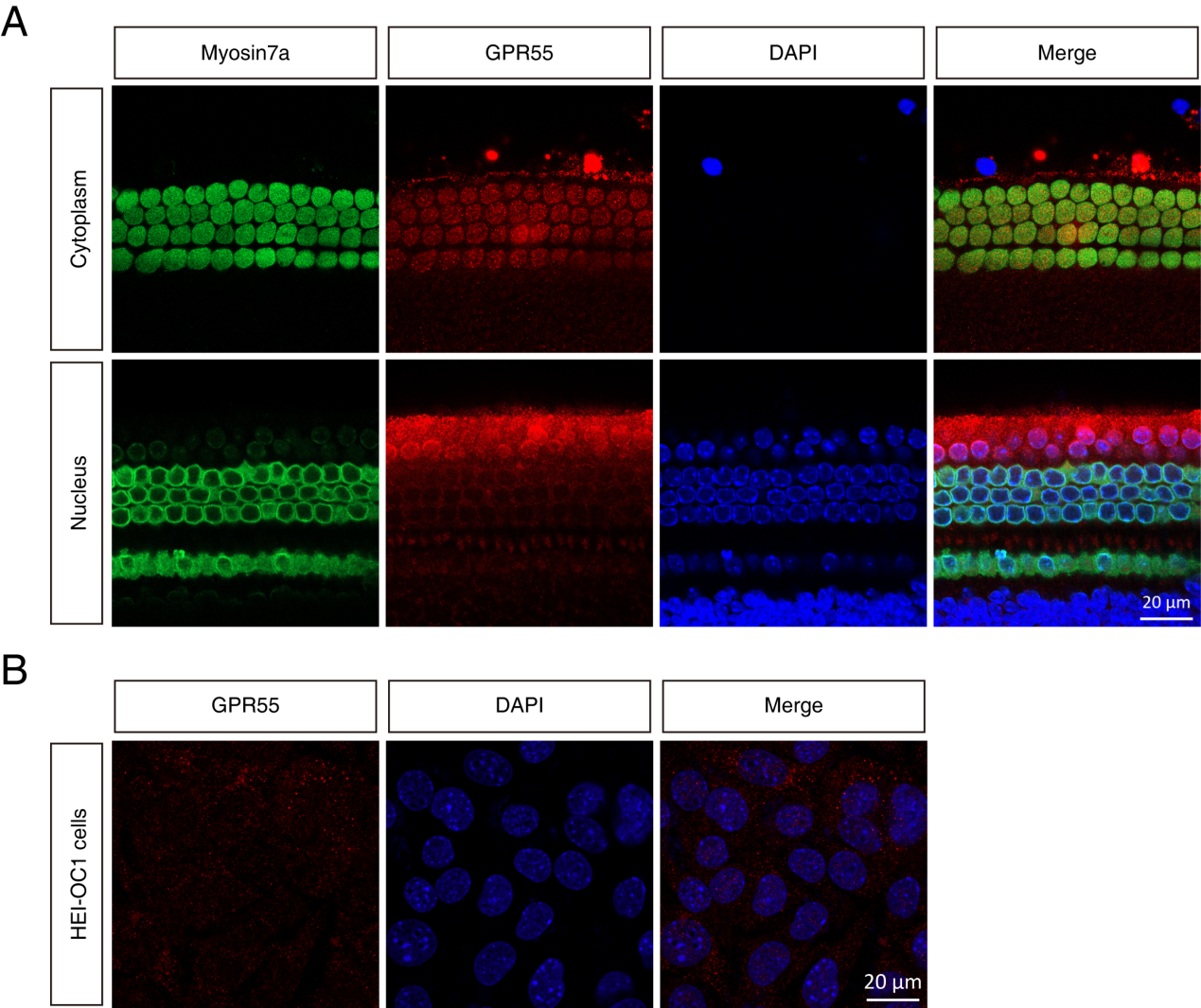


Fig. S1 The presence of GPR55 in HCs and HEI-OC1 cells. (**A**) Immunofluorescence staining revealed the distribution of GPR55 (red) in P3 mouse HCs (green), HCs were identified using anti-Myosin7a. (**B**) Immunofluorescence staining demonstrated the expression pattern of GPR55 (red) in HEI-OC1 cells. The nucleus was labeled with DAPI (blue).


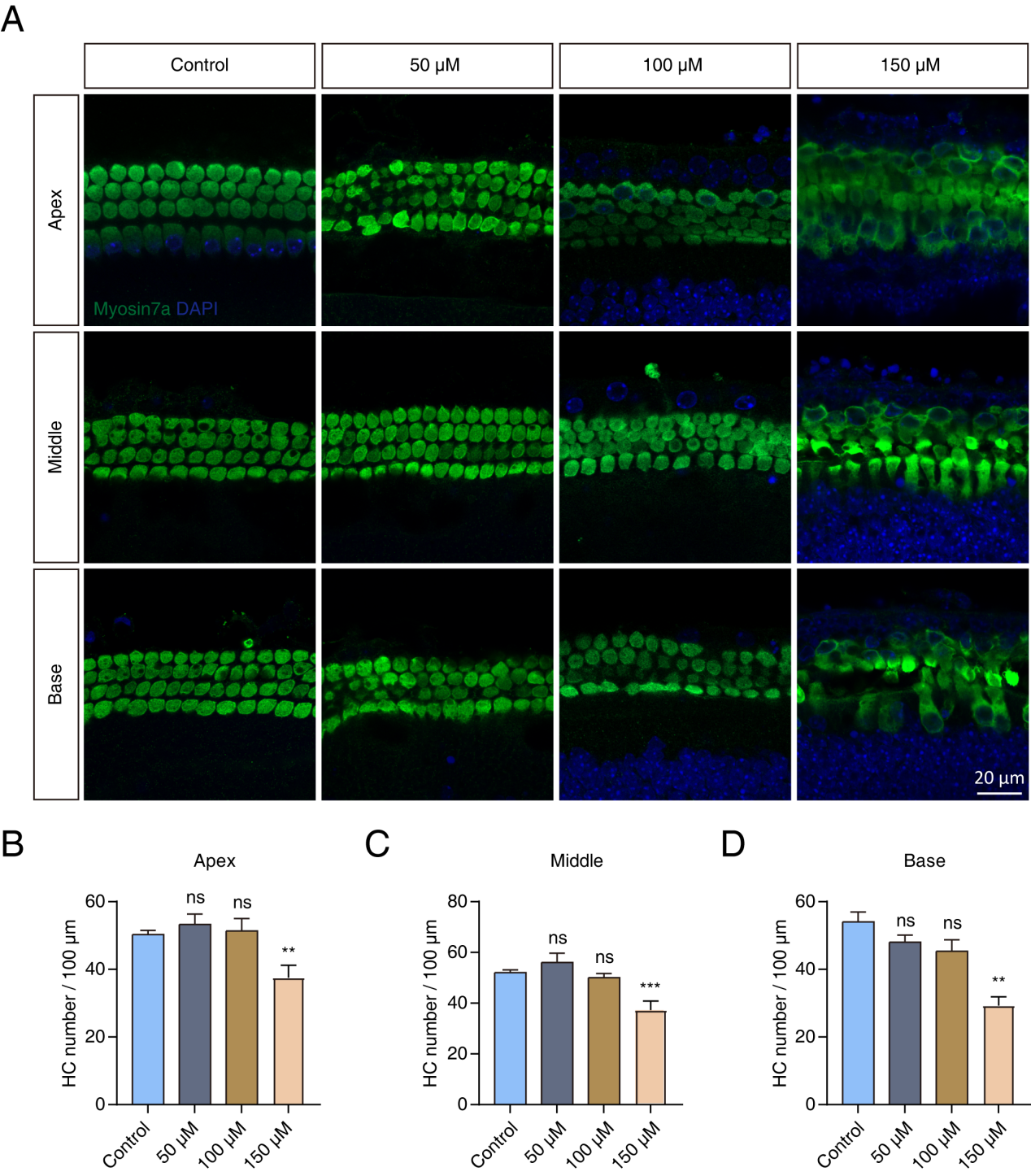


Fig. S2 CDDP exposure led to a dose-dependent loss of cochlear HCs. (**A**) Immunofluorescence images illustrated HCs in the apical, middle, and basal regions of cochleae from groups subjected to 0, 50, 100, or 150 μM CDDP for 24 h. (**B-D**) HC counts in apical, middle, and basal cochlear turns were recorded for every experimental group.


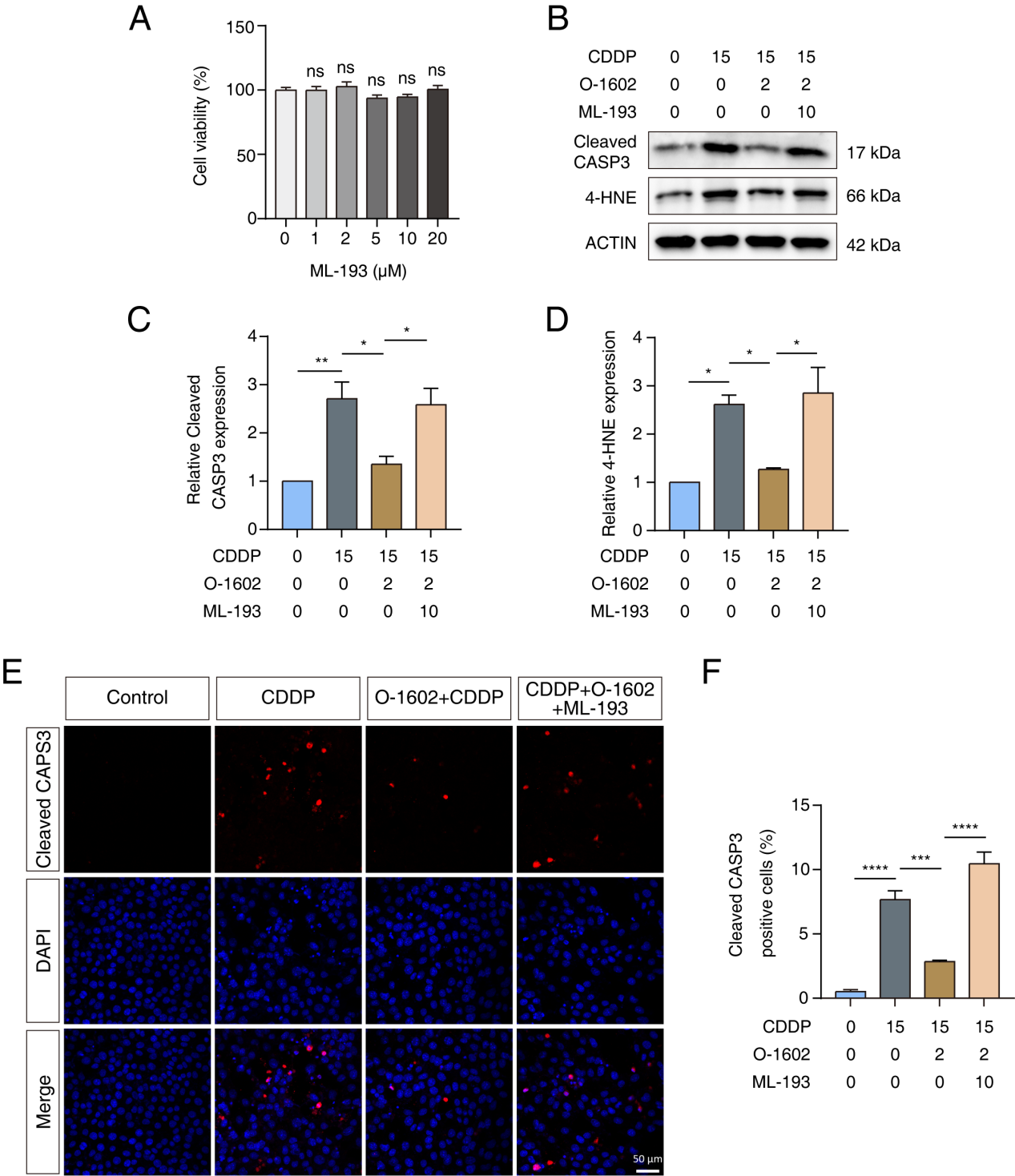


Fig. S3 The GPR55 inhibitor ML-193 negated the protective effect of O-1602 against apoptosis induced by CDDP in HEI-OC1 cells. (A) The CCK-8 assay was conducted to assess the cell viability of HEI-OC1 cells subjected to various concentrations of ML-193 (0, 1, 2, 5, 10, or 20 μM) for 24 h. (B, C) Representative Western blot images showed the expression of cleaved CASP3 and 4-HNE in different treatment groups. (E, F) Immunofluorescence analysis revealed the percentage of cleaved CASP3-positive cells in each group.


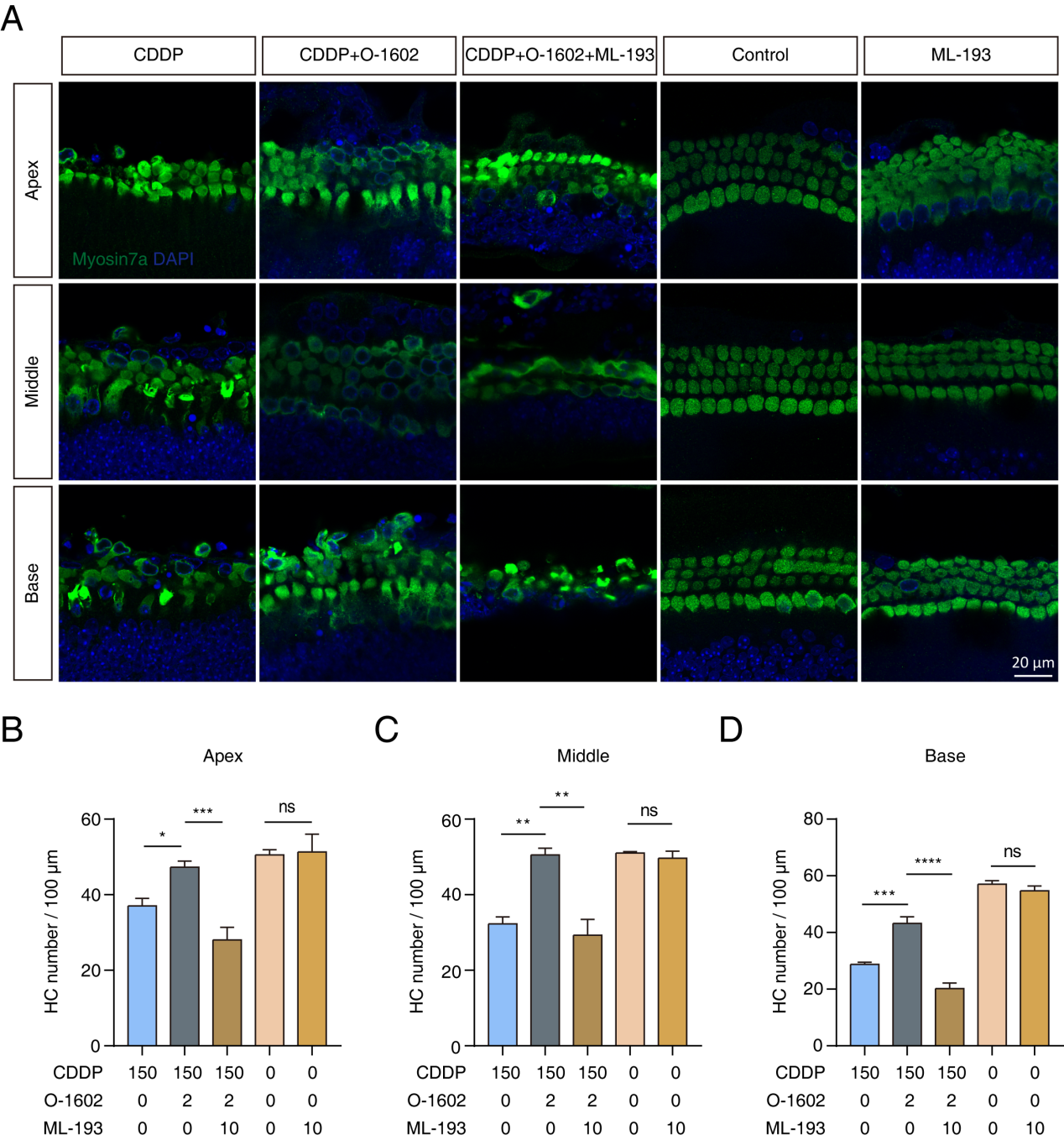


Fig. S4 The protective effect of O-1602 against CDDP-induced damage to HCs was eliminated by the GPR55 inhibitor ML-193. (**A**) Immunofluorescence images showed HCs in the apical, middle, and basal turns of the cochleae for each treatment group. (**B-D**) Quantification of HCs in the apical, middle, and basal turns of the cochlea was performed for each group.


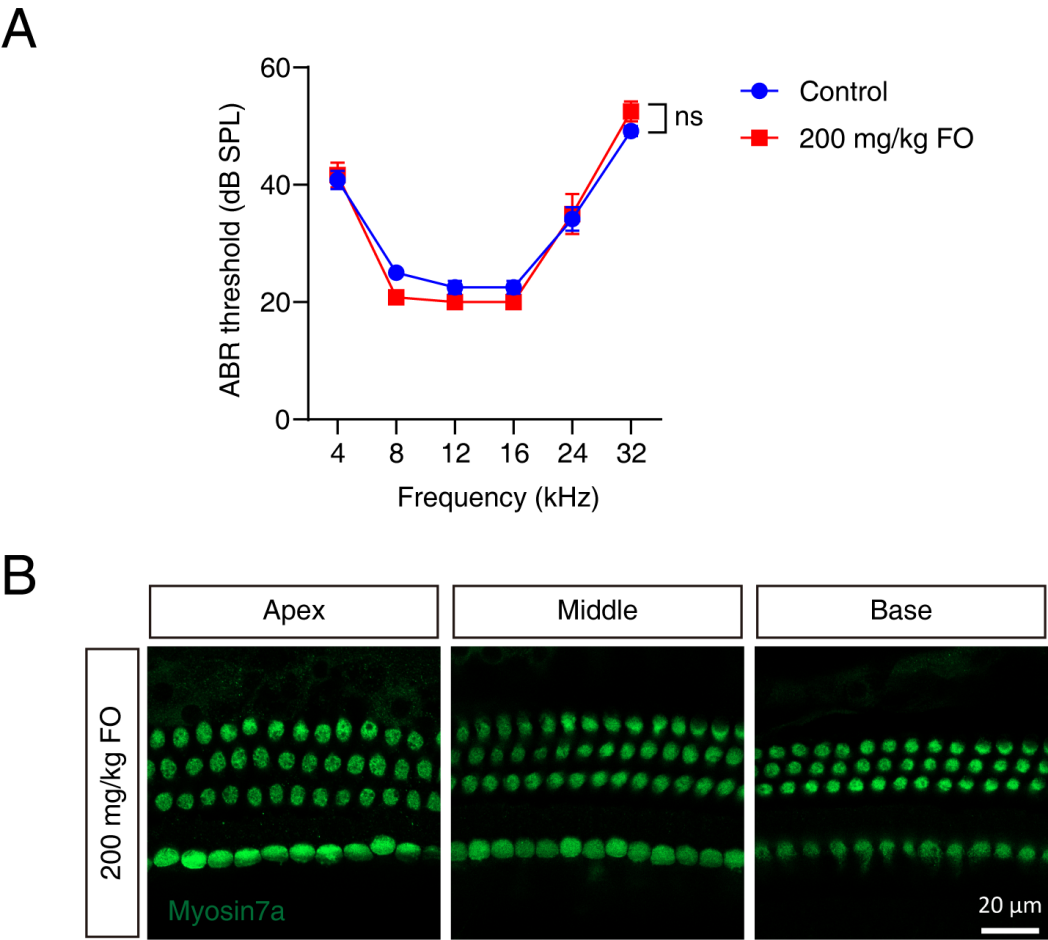


Fig. S5 The administration of FO alone did not induce ototoxicity. (A) ABR thresholds were assessed in both control and FO-treated mice (n = 6 per group). (B) Immunofluorescence analysis showed mouse cochlear HCs labeled with anti-Myosin7a (green).
